# An Orphan Gene Enhances Male Reproductive Success in *Plutella xylostella*

**DOI:** 10.1093/molbev/msae142

**Published:** 2024-07-11

**Authors:** Qian Zhao, Yahong Zheng, Yiying Li, Lingping Shi, Jing Zhang, Dongna Ma, Minsheng You

**Affiliations:** State Key Laboratory for Ecological Pest Control of Fujian/Taiwan Crops and College of Life Science, Fujian Agriculture and Forestry University, Fuzhou 350002, China; Ministerial and Provincial Joint Innovation Centre for Safety Production of Cross-Strait Crops, Fujian Agriculture and Forestry University, Fuzhou 350002, China; Joint International Research Laboratory of Ecological Pest Control, Ministry of Education, Fuzhou 350002, China; State Key Laboratory for Ecological Pest Control of Fujian/Taiwan Crops and College of Life Science, Fujian Agriculture and Forestry University, Fuzhou 350002, China; State Key Laboratory for Ecological Pest Control of Fujian/Taiwan Crops and College of Life Science, Fujian Agriculture and Forestry University, Fuzhou 350002, China; State Key Laboratory for Ecological Pest Control of Fujian/Taiwan Crops and College of Life Science, Fujian Agriculture and Forestry University, Fuzhou 350002, China; State Key Laboratory for Ecological Pest Control of Fujian/Taiwan Crops and College of Life Science, Fujian Agriculture and Forestry University, Fuzhou 350002, China; Ministerial and Provincial Joint Innovation Centre for Safety Production of Cross-Strait Crops, Fujian Agriculture and Forestry University, Fuzhou 350002, China; Joint International Research Laboratory of Ecological Pest Control, Ministry of Education, Fuzhou 350002, China; State Key Laboratory for Ecological Pest Control of Fujian/Taiwan Crops and College of Life Science, Fujian Agriculture and Forestry University, Fuzhou 350002, China; State Key Laboratory for Ecological Pest Control of Fujian/Taiwan Crops and College of Life Science, Fujian Agriculture and Forestry University, Fuzhou 350002, China; Ministerial and Provincial Joint Innovation Centre for Safety Production of Cross-Strait Crops, Fujian Agriculture and Forestry University, Fuzhou 350002, China; Joint International Research Laboratory of Ecological Pest Control, Ministry of Education, Fuzhou 350002, China

**Keywords:** orphan gene, sperm protein, male reproductive fitness, sperm competition, insulin

## Abstract

*Plutella xylostella* exhibits exceptional reproduction ability, yet the genetic basis underlying the high reproductive capacity remains unknown. Here, we demonstrate that an orphan gene, *lushu*, which encodes a sperm protein, plays a crucial role in male reproductive success. *Lushu* is located on the Z chromosome and is prevalent across different *P. xylostella* populations worldwide. We subsequently generated *lushu* mutants using transgenic CRISPR/Cas9 system. Knockout of Lushu results in reduced male mating efficiency and accelerated death in adult males. Furthermore, our findings highlight that the deficiency of *lushu* reduced the transfer of sperms from males to females, potentially resulting in hindered sperm competition. Additionally, the knockout of Lushu results in disrupted gene expression in energy-related pathways and elevated insulin levels in adult males. Our findings reveal that male reproductive performance has evolved through the birth of a newly evolved, lineage-specific gene with enormous potentiality in fecundity success. These insights hold valuable implications for identifying the target for genetic control, particularly in relation to species-specific traits that are pivotal in determining high levels of fecundity.

## Graphical abstract

**Graphical abstract:**
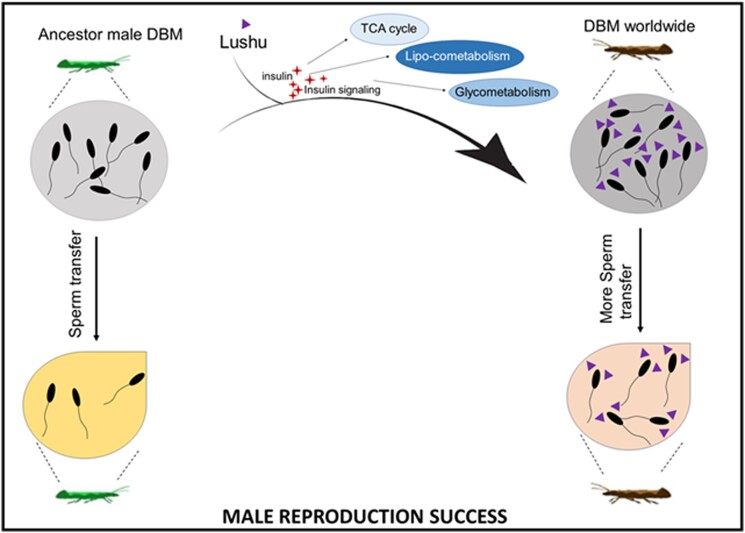

## Introduction

Effective pest population control requires the manipulation of crucial genes that are necessary for pest survival or reproduction, either through genetic control techniques or pesticide use. However, identifying these target genes and understanding their molecular mechanisms, while also minimizing negative impacts on nontarget organisms and the environment, requires extensive effort. Orphan genes, which exist in a unique evolutionary branch and lack homologous or corresponding genes in other organisms, often play crucial roles in species-specific adaptations or novel biological functions (Long et al. 2003; Kaessmann 2010; Ni et al. 2017). Consequently, orphan genes are prospective molecular targets in the development of sustainable pest control measures.

*Plutella xylostella*, also known as the diamondback moth (DBM), is a global pest that infests cruciferous vegetables (Brassicaceae), including cabbage, cauliflower, and rapeseed (You et al. 2013). The worldwide cost of managing crop damage caused by *P. xylostella* is estimated to be around $4 to 5 billion per year (Furlong et al. 2013). DBM is a notorious pest because of its high reproduction potential with over 20 generations per year and more than 100 eggs laid by each female moth following mating (Wei et al. 2010). A better understanding of the molecular basis for its strong fecundity can help reduce its reproductive capacity (Wang et al. 2021).

Acquisition of new/orphan genes is a fundamental evolutionary process that provides a potential source of phenotypic novelty to facilitate species adaptation and even speciation (Long et al. 2003; Ding et al. 2010; Yeh et al. 2012). The “out of testis” model (Kaessmann 2010) specifically suggests that novel genes are highly likely to originate in the testis and initially serve reproductive functions before being co-opted to perform various functions in other parts of the body (Marques et al. 2005; Kaessmann 2010; Yeh et al. 2012). In *Drosophila melanogaster*, previous studies showed that the de novo genes were frequently X-linked and exhibit testis-biased expression (Levine et al. 2006; Begun et al. 2007). Similarly, new genes with male-biased expression patterns have also been identified in plants (Wu et al. 2014; Ni et al. 2017), insects (Betrán et al. 2002; Dorus et al. 2008), and mammals (Emerson et al. 2004; Marques et al. 2005), and contribute to variations in male fertility (Yeh et al. 2012). Several studies so far have confirmed that new genes can confer male-related fitness advantages by affecting male courtship (Dai et al. 2008) or male fertility-related functions (Loppin et al. 2005; Kalamegham et al. 2007; Lange et al. 2021) such as sperm production (Kalamegham et al. 2007), individualization, motility (Heinen et al. 2009; Ding et al. 2010), and sperm axonemes elongation (Lange et al. 2021). Therefore, characterization of phenotypes associated with new/orphan genes that affect male-related functions is especially important for understanding the male reproductive adaptation and fitness.

Due to the lack of phylogenetic conservation in orphan genes, their origin remained elusive. Over the last few decades, studies have provided insight into diverse mechanisms underlying the emergence of orphan genes. For example, de novo originated genes were interpreted as having evolved from previously noncoding sequences (Levine et al. 2006; Begun et al. 2007; Wu et al. 2011; Lange et al. 2021). Duplicated genes that acquire a novel function can undergo radical divergence, making them unrecognizable compared to their homologs (Domazet-Loso and Tautz 2003; Tautz and Domazet-Loso 2011). Nondeleterious frameshift mutations following gene duplication have been shown to potentially generate novel genes, as evidenced in study of mouse (Okamura et al. 2006). Additionally, sequences derived from transposable elements (TEs), such as Alu repeats in primates, may integrate into existing human genes, often resulting in the formation of new exons (Makalowski et al. 1994). TE insertions have also been proposed to contribute to the creation of two mouse genes (Nekrutenko and Li 2001). Besides, numerous orphan genes with unknown origins have been recognized, and these genes make up a specific proportion of the genome (Toll-Riera et al. 2009; Sun et al. 2015).

Here, we have provided functional evidence that the novel gene *lushu* was responsible for conferring male reproductive success in *P. xylostella*. Our study revealed that *lushu* encoded a sperm protein. Males with a deficiency in the Lushu exhibited lower mating rate, earlier death, and low sperm competition. Additionally, we noted a marked decrease in sperm storage in the bursa after mating with mutant males. This could potentially be one of the factors contributing to the diminished performance in sperm competition. Moreover, our findings indicated that the absence of Lushu leads to disruptions in glucose homeostasis, lipid metabolism, TCA cycle, and insulin signaling pathways in males, suggesting a correlation between the functions of *lushu* and energy metabolism-related pathways. This investigation provides evidence on how an orphan gene infiltrates an existing genetic regulatory network to contribute to an adaptive function.

## Results

### The Orphan Genes are Potentially Associated With Male Reproductive Functions

**Fig. 1.**
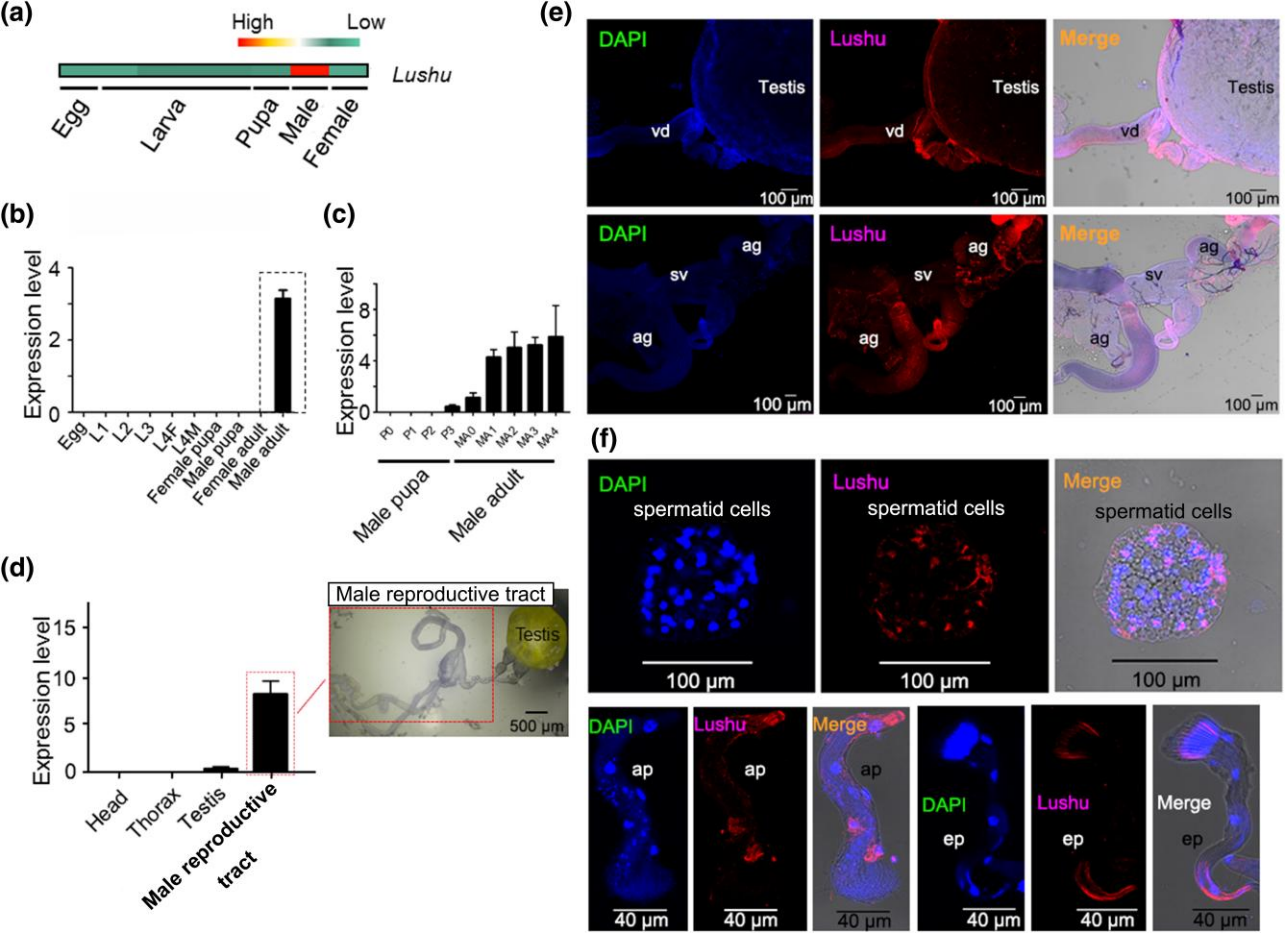
A sperm protein encoded by *Lushu* in *P. xylostella.* a) *Lushu* was highly expressed in male adult based on RNA-Seq data. RNA-Seq was conducted for newly laid egg, larvae (from first- to fourth-instar larvae, and the sample of fourth instar larvae is an equal mixture of males and females), pupa (>2 d, equal mixture of males and females), virgin male and female adults. b) Expression patterns of *lushu* at different developmental stages. Expression level represents the relative expression level using *RIBP* as the control gene for qRT-PCR. L1, L2 and L3, indicate first, second, and third instar larvae, respectively; L4F, fourth instar female larvae; L4M, fourth instar male larvae. Term “Male/Female pupa” refers to the developmental stages spanning from first- to fourth- day after pupation for males/females (samples of an equal mixture of each day). Male/Female adult includes the developmental stages from first- to fifth- day male/female adults (samples of an equal mixture of each day). The relative expression level is represented as the mean ± SD (*n* = 4). c) *Lushu* started to express at the later stage of male pupa and reached the highest expression level at male adult stage. P0–P3, first- to fourth- day male pupa; MA0–MA4, first- to fifth-day male adults. d) Expression patterns showed that *lushu* was highly expressed in male reproductive tract except testes including accessory gland, vas deferens and seminal vesicles. Expression level represents the relative expression level using *RIBP* as the control gene for qRT-PCR. e) Lushu was located in gonad, vas deferens, accessory gland, and seminal vesicles verified by immunofluorescence. ag, accessory gland; vd, vas deferens; sg, silkgland. Reproductive tissues were dissected from 1d-old male adults. (f) Lushu was located on sperm bundles dissected from testis. Ap, apyrene sperm bundles; ep, eupyrene sperm bundles.

To identify genes related to *P. xylostella* specific reproduction, we searched for the orphan genes based on comparative analysis (Wu et al. 2011; Sun et al. 2015) with nonredundant protein sequences (*nr* database), which includes almost all the predicted proteins. According to our criteria, these orphan proteins do not possess any homologous proteins in other species. Totally, we identified 1,100 orphan genes in the *P. xylostella* genome (supplementary table S1 and supplementary fig. S1a, Supplementary Material online). Comparing to other genes, orphan genes exhibit distinct characteristics such as shorter gene length, fewer exons, higher GC content, and a higher isoelectric point (supplementary fig. S1b, Supplementary Material online). To explore potential functions of these orphan genes, we analyzed their expression patterns using RNA-Seq. Results showed that 837 of the 1,100 orphan genes (76.1%) were expressed (RPKM > 2) and 61 of these exhibited high transcript abundance (RPKM > 100) in at least one developmental stage (supplementary fig. S2a, Supplementary Material online). Among these 61 orphan genes, we found seven genes that were specifically highly expressed in male adult (supplementary fig. S2a, Supplementary Material online). Using quantitative reverse transcription polymerase chain reaction (qRT-PCR), these genes were further validated to be specifically highly expressed in adult male reproductive tract (Fig. 1a–c, supplementary fig. S2b and c, Supplementary Material online). Therefore, we proposed that these seven orphan genes may have evolved certain functions that were related to male reproductive fitness.

### Lushu Functions as a Sperm Protein and is Delivered to Females in High Abundance

We focused a specific gene, named *lushu* (Px012388.1), which exhibits high expression in adult males (Fig. 1a–c), particularly in the male reproductive tract (Fig. 1d). Molecular cloning showed that *lushu* was 522 bp in length without intron (supplementary fig. S3a, Supplementary Material online), and contained a 20-aa signal peptide at its N-terminus (supplementary fig. S3b, Supplementary Material online). This indicated that its polypeptides could be processed by a signal peptidase to release a soluble fragment into the male gonad. Through immunofluorescence assays, we observed its presence in the testes, male accessory gland, vas deferens, and seminal vesicles (Fig. 1e, supplementary fig. S3c and d, Supplementary Material online). Particularly, we identified its localization on sperm bundles (Fig. 1f, supplementary fig. S3e, Supplementary Material online), suggesting its role as a sperm protein. We then characterized the sperm proteins of *P. xylostella* (supplementary fig. S4a and b, Supplementary Material online). Using tandem mass spectrometry (MS/MS) to analyze mixed eupyrene and apyrene sperms, we identified 915 sperm proteins from three sets of replicates with high confidence, meaning that they were found in all three replicates (supplementary table S2, Supplementary Material online). Among these proteins, Lushu was a high-abundance protein (top 5% of content, Table 1, supplementary fig. S4c and d, Supplementary Material online). To further eliminate the possibility of it being a seminal fluid protein (SFP), we employ multiple criteria. Firstly, according to previous studies, SFPs have been shown to be proteins that, after mating, become significantly less abundant in the male accessory glands (MAGs) or the ejaculatory duct, but more abundant in the female reproductive tract (Sepil et al. 2019 ; Hurtado et al. 2022) . It was found that the abundance of Lushu does not exhibit a significant decrease in the male accessory gland (MAG) after mating (NSAF = 0.00263 before mating *vs.* NSAF = 0.00256 after mating, Student's *t*-test, *P* = 0.11), although it showed high abundance in female bursa (Table 1). Secondly, a previous study of *P. xylostella* identified seminal fluid proteins (SFPs) based on proteome analysis, but Lushu was not among these proteins identified (Wu et al. 2023). Thirdly, sperm samples used in immunofluorescence assays were collected from testes (Fig. 1f), seminal vesicles, and vas deferens (supplementary fig. S3e, Supplementary Material online), while the SFPs were proved to be in male accessory glands (MAGs). Our findings based on expression evidence, immunofluorescence assay, structure analysis, and mass spectrometry analysis indicated that *lushu* gene encodes a sperm protein.

**Table 1:** Lushu showed high abundance as a sperm protein and is delivered to female with high abundance based on LC-MS/MS analysis

Tissue	Peptides	NSAF	Ranking
*Seminal vesicles*	15	0.0025	79/915
Bursa	7	0.0104	14/692

Relative protein abundance was assessed by calculating the normalized spectral abundance factor (NSAF). Peptides represented the peptide number of Lushu identified in the LC-MS/MS analysis. Ranking indicated the rank of protein abundance for Lushu.

To further verify whether Lushu was delivered to females, we conducted further characterization of the fluid proteins in the bursa of females within 30 min after mating using LC-MS/MS analysis. Our results indicated that Lushu protein was highly abundant (Table 1; supplementary table S3 and supplementary fig. S4e and f, Supplementary Material online). Considering low transcriptional expression of the *lushu* gene in females (Fig. 1b), it is reasonable to infer that the prevalence of Lushu in females is due to transfer from males.

### Constructing Lushu Mutants Using CRISPR/Cas9 System

**Fig. 2.**
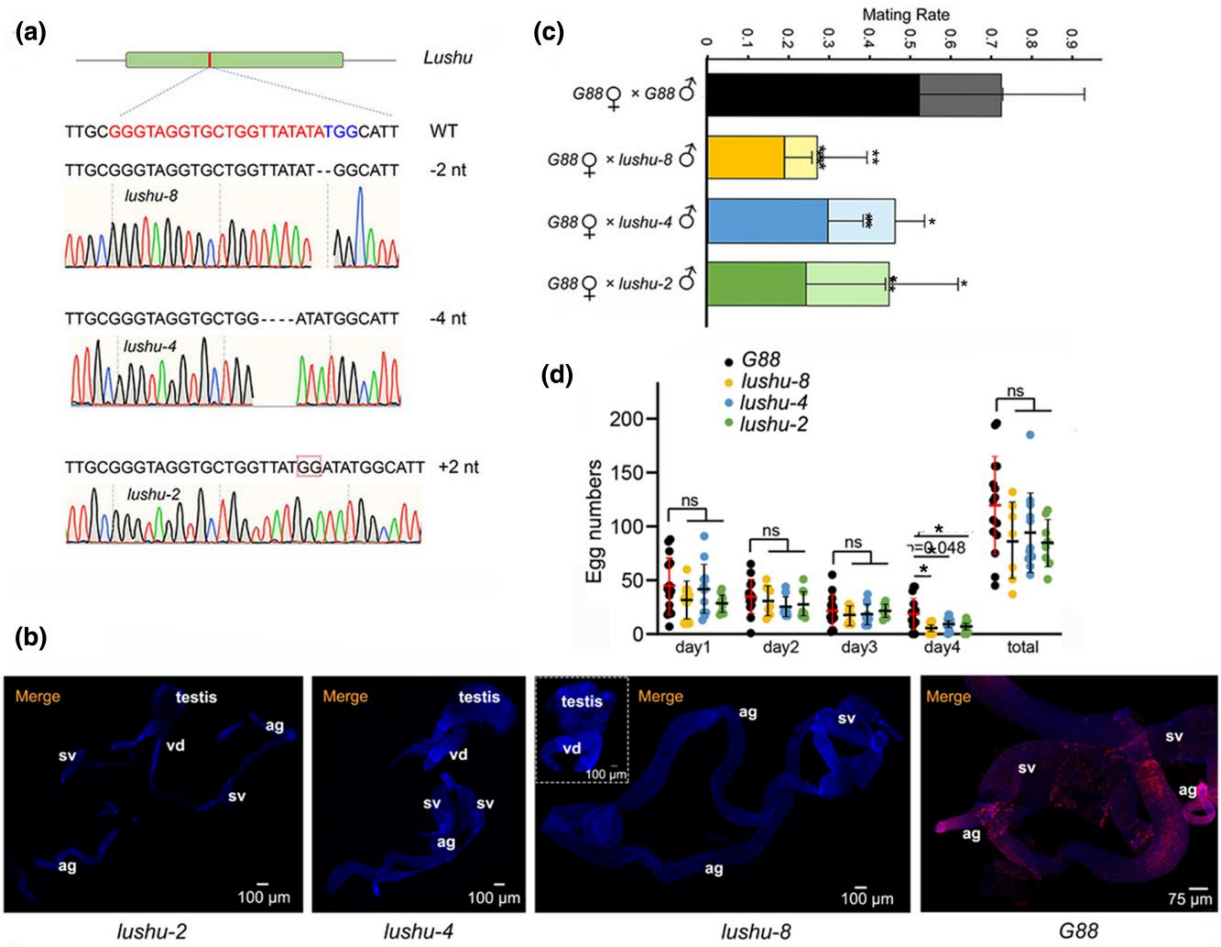
Mutagenesis of *Lushu* using CRISPR/Cas9. a) Representative sequences from wild type (*G88*) strain and the *lushu-null* mutant strain showing a 2 bp- deletion (*lushu-8*), a 4 bp- deletion (*lushu-4*), and a 2 bp- insertion (*lushu-2*). b) Tissues of reproduction from male *G88* and *lushu-null* DBM strains were incubated with Lushu polyclonal antibody and HRP-Goat Anti-Rabbit IgG. We cannot detect the Lushu (red) in *lushu-null* males, suggesting that Lushu was successfully knocked out in the mutant males. c) Comparison of mating rate between males of *G88* and *lushu-null*. And mating within 30 min was indicated in darker color for each mating pair. Statistics were obtained from 10 biological replicates, with each replicate containing six mating pairs. The mating rate is represented as the mean ± SD (*n* = 10). Asterisks indicate significant difference: *P < 0.05, **P < 0.01 (Student's *t*-test). The mating rate of *G88* males within 30 min was significantly higher than that of *lushu-8* males (*P* = 0.0072), *lushu-4* males (*P* = 0.0119), and *lushu-2* males (*P* = 0.0197). Additionally, the overall mating rate of *G88* males within 3 h was significantly higher than that of *lushu-8* males (*P* = 0.0013), *lushu-4* males (*P* = 0.0399), and *lushu-2* males (*P* = 0.0304). d) Estimation and comparison of male fertility in terms of egg number between males of *G88* and *lushu-null* under one-to-one mating. Totally 15 replicates were prepared for this analysis. The egg number is represented as the mean ± SD (*n* = 15). The statistical analysis was conducted using Student's *t*-test, and “ns” indicated nonsignificance. The number of eggs laid by females mating with *G88* males was significantly higher than those mating with *lushu-8* males (*P* = 0.042), *lushu-4* males (*P* = 0.048), and *lushu-2* males (*P* = 0.047).

To address its functions, we used the CRISPR/Cas9 approach to knockout Lushu protein and obtained three mutant lines, each carrying a specific mutation resulting in a 2-bp deletion, a 4-bp deletion, and a 2-bp insertion in the gene. Mutations in randomly selected representative offsprings were detected by PCR and sequencing using gene-specific primers, confirming the occurrence of mutations in both male and female individuals (Fig. 2a). These mutant strains were individually designated as *lushu-8*, *lushu-4*, and *lushu-2*, and were subsequently verified via immunofluorescence assay and tandem mass spectrometry (MS/MS) (Fig. 2b; supplementary fig. S5a–c, Supplementary Material online). These results indicated that we successfully generated mutant lines. Furthermore, candidate edited mutations were identified through whole genome re-sequencing. Firstly, CRISPR-edited sites were predicted using Cas-OFFinder algorithm, which revealed 112 potential edited sites (supplementary table S4, Supplementary Material online). Subsequently, these predicted CRISPR-edited sites were compared with the variations found in the mutants identified by whole-genome re-sequencing. If the overlapped sites coincided with the variations of *G88* identified by whole-genome re-sequencing, they are excluded. The findings indicated that, besides the CRISPR-edited sites on *lushu* (supplementary fig. S5d, Supplementary Material online), no other CRISPR-edited sites were detected (supplementary table S5, Supplementary Material online).

### Lushu Confers Reduced Mating Rate and Shorter Adult Lifespan of Adult Males

We investigated whether *lushu-null* males exhibited defects in male reproductions including mating behaviors and the offspring. We monitored the mating rate within a three-hour period, during which most of the adult pairs successfully mated (Zou et al. 2022). We found a significant decrease in the mating rate for *lushu-null* males (Fig. 2c; supplementary Video S1, Supplementary Material online). Nevertheless, among these successfully mated pairs, no significant morphological changes (supplementary fig. S6a, Supplementary Material online) or hatching rate differences (supplementary fig. S6b, Supplementary Material online) were observed among the offspring eggs in *lushu-null* males, indicating that Lushu is not a core sperm protein responsible for egg fertilization and development. Following that, we proceeded to monitor the offspring count during the subsequent four days after mating, which encompasses almost the entire egg-laying period of *P. xylostella.* Results showed that there was no statistically significant difference in progeny numbers during the first three days or in total progeny numbers (Fig. 2d). These findings suggested that Lushu was not a core component of spermatogenesis responsible for rendering male fertility. To provide some context for our observation, we summarized the spermatogenesis in *P. xylostella*. In the testes, spermatogenesis begins with round spermatid cells undergoing an elongation process to generate bundles of nearly mature spermatozoa (Cai et al. 2008). We did not observe any noticeable morphological abnormality during spermatogenesis in *lushu-null* males (supplementary fig. S6c, Supplementary Material online), and the sperm vitality rate remained stable (supplementary table S6, Supplementary Material online). These findings also supported the notion that Lushu was not a core component responsible for spermatogenesis. To be noticed, we found that there were significantly fewer eggs on the fourth day (Fig. 2d). Further investigation revealed that this may be a result of the *lushu-null* males experiencing earlier death. Our findings indicated that *lushu-null* male adults exhibited a notably shortened lifespan, approximately 2 to 3 d shorter, when compared to *G88* male adults (Student's *t*-test, *P* = 0.0000; supplementary fig. S7, Supplementary Material online). These results suggested that males lacking Lushu protein undergo earlier death.

### Lushu is Essential for Male Sperm Competition

**Fig. 3.**
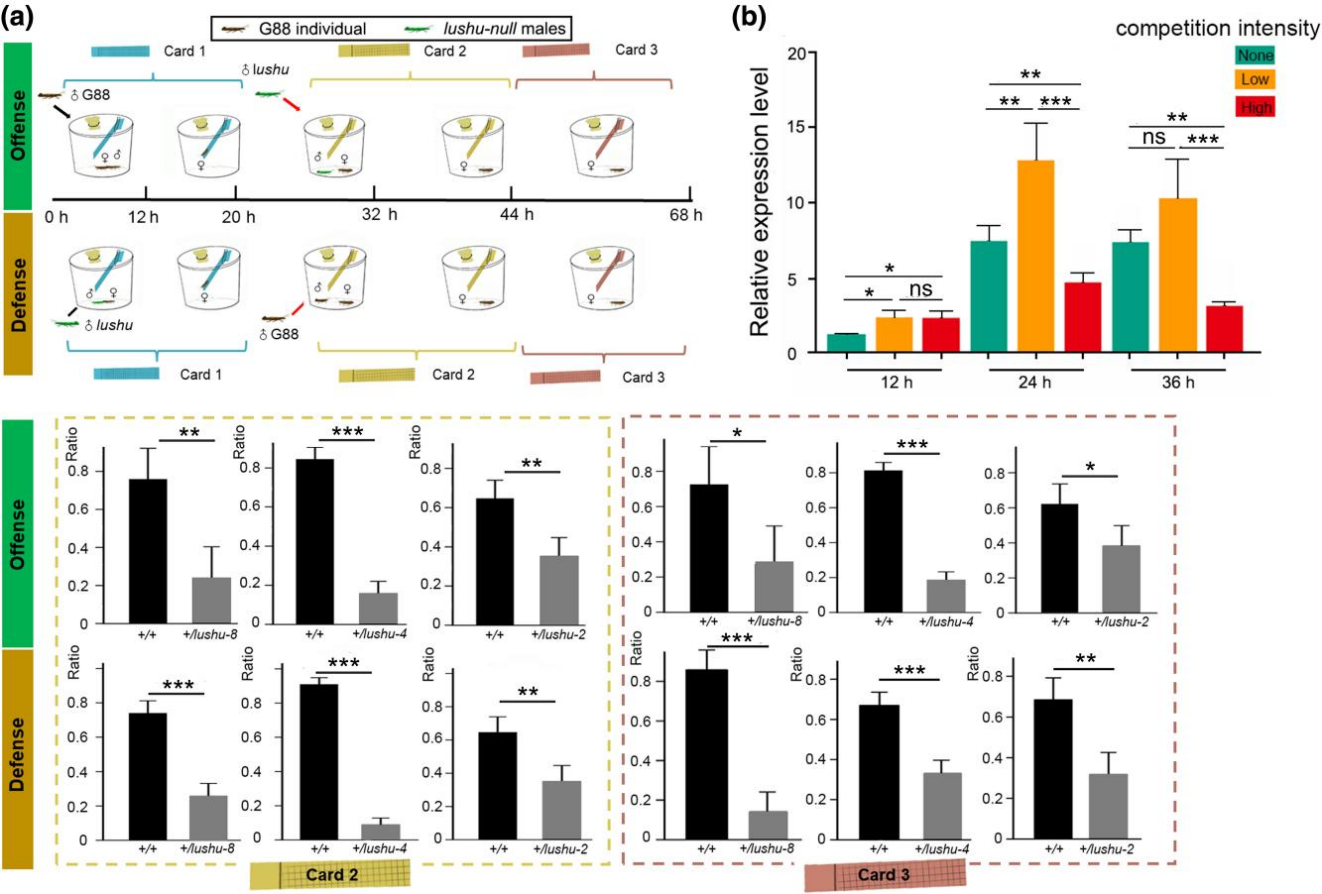
Evidence for involvement of Lushu in sperm competition. a) Sperm competition conferred by *lushu*. The competition index (Ratio) calculated in this study is designated as P2 in the Materials and Methods. Given the ratios of offspring genotypes observed, successful paternity by *G88* or *lushu-null* males can be calculated and illustrates the differences in sperm competition between genotypes. Ratio is represented as the mean ± SD (*n* = 3). Asterisks indicate significant differences: *P < 0.05, **P < 0.01, and ***P < 0.001 (Fisher's Exact Test). b) Expression patterns of *lushu* along different competition gradients. The relative expression level is represented as the mean ± SD (*n* = 4) for qPCR analysis. Asterisks indicate significant differences: *P < 0.05, **P < 0.01, and ***P < 0.001 and ns indicates no significant difference (Student's *t*-test).

In *P. xylostella*, females often mate with more than one male (Song et al. 2014). It determines the pattern of sexual selection and sexual conflict. This behavior results in competition between the sperm of different males (Milkmann and Zeitler 1974; Griffiths et al. 1982; Gallup et al. 2006; Tan et al. 2011) and difference in fertilization success (Manier et al. 2010; Droge-Young et al. 2016). Fertilization success in sperm competition is often determined by estimates of the proportion of offspring sired by the first (P1) or second (P2) males that mates with the same female (Singh et al. 2002). Considering *P. xylostella* belongs to the clade with last male sperm precedence (with a significantly higher P2 value) (Simmons and Siva-Jothy 1998), we performed two kinds of sperm-displacement assays, termed “offense” and “defense” (Fig. 3a, see methods). For the offense assay, we evaluated the ability of sperms from *lushu-null* males to displace or inactivate the sperms from *G88* males (the proportion of offspring with the genotype +/*lushu*). As the results showed, we detected significantly higher number of progenies from the *G88* males (Fig. 3a). For the defense assay, we evaluated the ability of sperms from *lushu-null* males to resist displacement or inactivation by the sperms from *G88* males and also found significantly higher number of progenies fathered by *G88* males (the proportion of offspring with the genotype +/*+*) (Fig. 3a). Collectively, males lacking a functional Lushu protein exhibit poor competitive ability even if they are the last male to mate (P2 value is significantly lower) (Fig. 3a), indicating significantly lower sperm competition in *lushu-null* males.

In addition, we analyzed the expression level of *lushu* along a competition gradient. Previous studies proved that different competition intensity effectively induce differential responses in seminal fluid proteins (Dai et al. 2008). We set the same competition scenarios that was previously reported in *D. melanogaster* with individually housed males experience no competition, pairs experiencing low competition, and groups of eight experiencing high competition. The results revealed that expression of *lushu* continues to increase from 12 to 24 h and remains constant from 24 to 36 h when males experiencing no competition (Fig. 3c). The expression profile of *lushu* significantly increased from noncompetition to low competition intensity, while it decreased notably as competition intensity increased (Fig. 3c). This indicated that the expression of *lushu* was plastically adjusted based on the intensity of sperm competition.

### Lushu Enhance the Ability to Accumulate Sperm into Storage

**Fig. 4.**
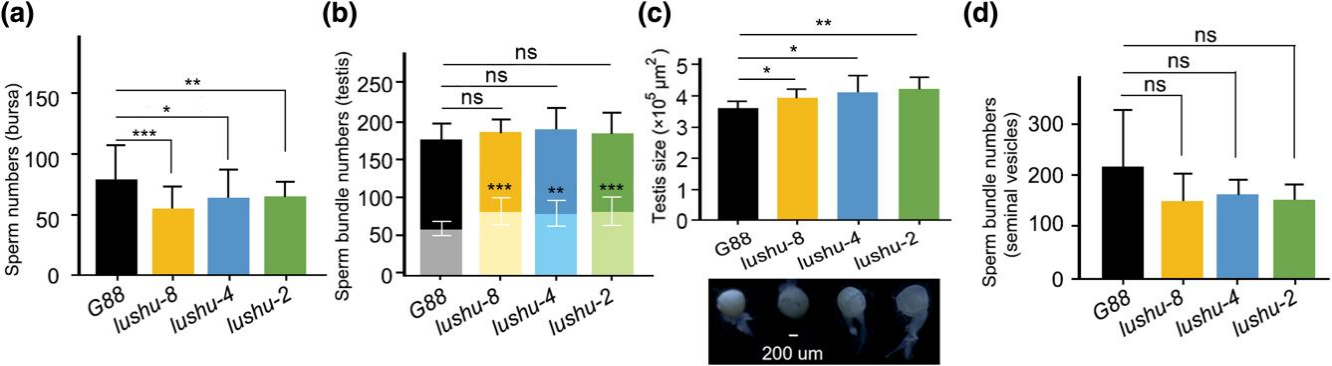
Sperm numbers and testis size comparison. a) Comparison of sperm numbers in the Bursa after 30 min of mating with wild-type and mutant males. The sperm numbers were represented as the mean ± SD (*n* = 40). Student's *t*-test was utilized to conduct the statistical analysis. b) The comparison of sperm bundle numbers in the testes of 1-day-old wild-type and mutant males, before and after mating, is illustrated. The counts of sperm bundles are expressed as the mean ± SD (*n* = 25). Within each bar graph, the darker shade signifies the sperm bundle count before mating, while the lighter shade indicates the sperm bundle count after mating. The statistical analysis of sperm bundle counts in the testes of *G88* versus *lushu-null* males was conducted using the Student's *t*-test. c) Testis size comparison between *G88* males and mutant males. The testis size is represented as the mean ± SD (*n* = 15). Statistical analysis was conducted using Student's *t*-test. (d) Comparison of sperm bundle numbers in seminal vesicles of wild-type and mutant males at 1 d old. The sperm bundles numbers were represented as the mean ± SD (*n* = 25). Asterisks indicate significant differences: *P < 0.05, **P < 0.01, and ***P < 0.001 and ns indicate no significant difference.

We proceeded to collect eggs after 68 h for the sperm-displacement assays. The results revealed a significantly decreased in the number of eggs in “offense” assays (where *lushu-null* males mated last) compared to “defense” assays (where *G88* mated last) (supplementary fig. S8a, Supplementary Material online). This decrease could be attributed to the lower sperm accumulation into storage during the same mating period by mating with *lushu-null* males compared to *G88*, resulting in a reduced number of offspring as the sperm were depleted. To confirm this hypothesis, we separated the female and male adults 30 min after mating. Previous studies showed that 30 min was adequate for sperm transfer from males to females in *P. xylostella* (Wu et al. 2023). The number of eggs laid were then recorded. Results indicated that the quantity of eggs laid following mating with *lushu-null* male was significantly lower than that with *G88* males (supplementary fig. S8b and c, Supplementary Material online). At the same time, we examined the stored sperms when females mated with *G88* males as opposed to those mated with *lushu-null* males. The results indicated that females mated with *G88* males had a significantly higher sperm count compared to those mated with *lushu-null* males (Fig. 4a). This suggested that the presence of Lushu enables more efficient sperm storage in females, leading to successful sperm competition.

The decreased number of sperm in bursa could be attributed to either a lower initial sperm counts or a reduced number of sperm migrating into the bursa. Therefore, we counted the number of sperm bundles in testes before and after mating. The results indicated that there was minimal variation in the number of sperm bundles within the testes of *lushu-null* males (Fig. 4b), despite their enlarged testes, when compared to *G88* males before mating (Fig. 4c; supplementary fig. S8c, Supplementary Material online). After mating, the testes of mutant males had more sperm bundles (Fig. 4b). And approximately 36.5% of sperm bundles remained in the testes in *G88*, while *lushu-2*, *lushu-4*, and *lushu-8* males retained around 44.8%, 43.8%, and 44.9% of sperm bundles in the testes after mating, indicating a significantly higher retention rate compared to the wild-type (supplementary fig. S8d, Supplementary Material online). Furthermore, we observed a slightly lower number of sperm in the seminal vesicles of *lushu-null* males, although this difference was not statistically significant (Fig. 4d). These findings suggested that the deletion of Lushu may impact sperm transfer, leading to reduced accumulation of sperm in storage.

### Putative Interaction Network of *lushu*

As expected, as an orphan gene, *lushu* showed no significant association with any GO term in a DAVID search (https://david.ncifcrf.gov/). To reveal the effect of *lushu* on the transcriptome, we compared the mRNA from *G88* and *lushu-8*. Based on the annotation of the DBM genome (You et al. 2013), we detected 514 genes exhibiting significantly differential expressions (DEGs, adjusted *P* < 0.05; |log_2_^change fold^| > 1.5) between the G88 and *lushu-8* male *P. xylostella* (supplementary table S7, Supplementary Material online). Of these, 356 DEGs matched the Gene Ontology (GO) database, with 241 genes represented by 99 significant GO terms (supplementary table S8, Supplementary Material online). These DEGs were significantly enriched for metabolism-related biological processes including metabolic process, organonitrogen compound metabolic process, and small molecule metabolic process (supplementary fig. S9, Supplementary Material online, Fisher's exact test, FDR < 0.05). Furthermore, these DEGs were also enriched for KEGG pathways such as metabolic pathways, fatty acid metabolism, glycolysis/gluconeogenesis, and TCA cycle (supplementary fig. 10, Supplementary Material online).

**Fig. 5.**
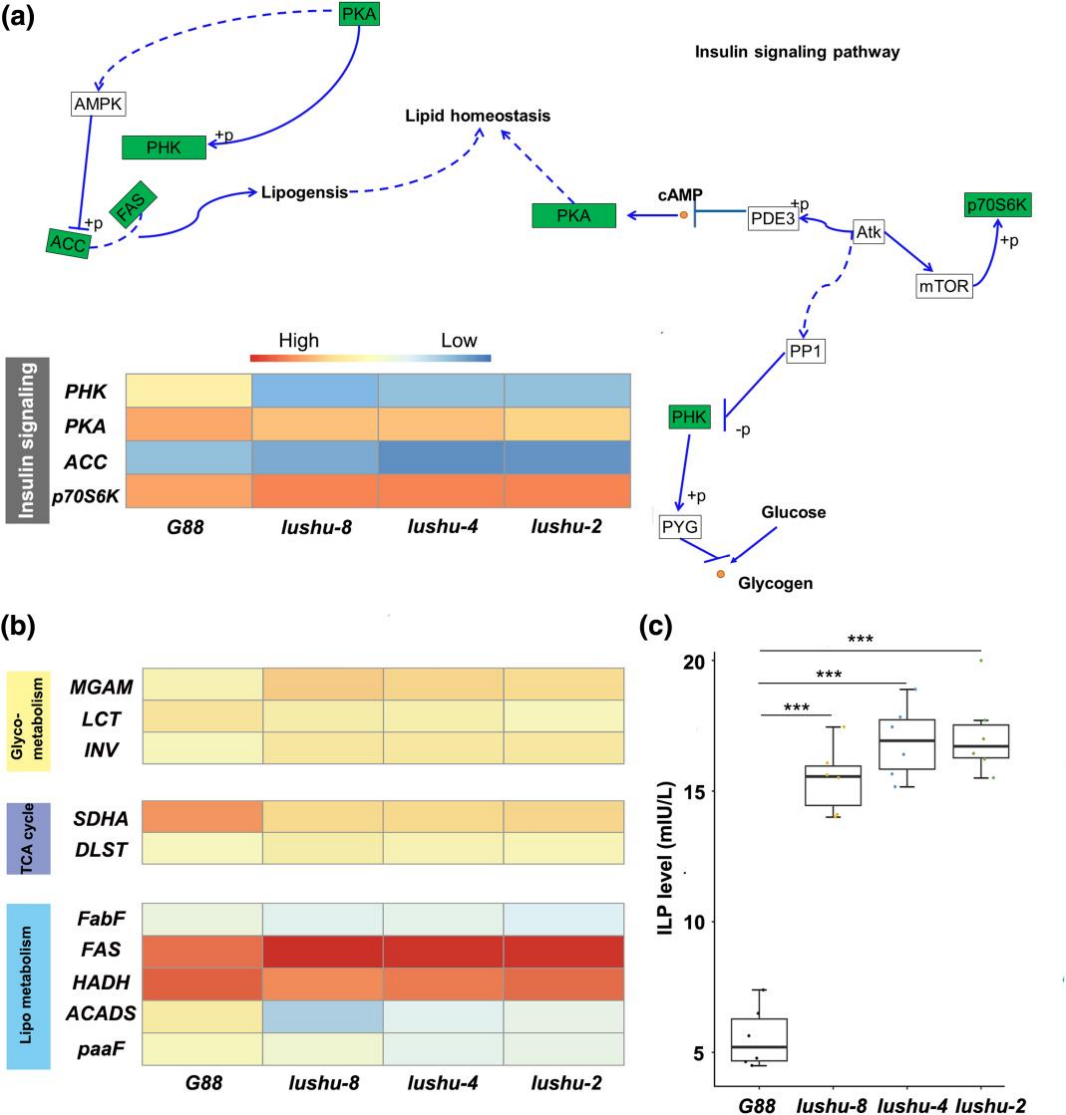
Genetic basis of metabolic pathways in which *lushu* might function. a) A proposed model for an insulin signaling pathway in which *lushu* might function in *P. xylostella*. Genes with disordered expression patterns were marked as green in *lushu-null* males. The heatmap illustrated the relative expression levels of these genes with disordered expression patterns, assessed using *RIBP* as the control gene for qRT-PCR. Relative expression level is represented as the mean (*n* = 4) in heatmap. Genes that showed significant differential expression between *G88* and all other three *lushu-null* strains were shown (Student's *t*-test). b) Key genes located on pathways controlling galactose metabolism, TCA cycle, and fatty acid metabolism are proved to be differently expressed between G88 and *lushu-null* males using qPCR. Genes that showed significant differential expression between *G88* and all other 3 *lushu-null* strains were shown (Student's *t*-test). c) ILP levels in *G88* male and *lushu-null* males. Significant differences were analyzed using Student's *t*-test (*n* = 6).

Notably, we found that many of these DEGs were significantly enriched in the energy metabolism-related pathways (supplementary fig. S9, Supplementary Material online). For example, we identified four DEGs enriched in the insulin signaling pathway (Fig. 5a), which is regulated by the hormone responsible for glucose homeostasis (Bogan 2012). Insulin acts as a key regulator of glucose homeostasis (Bogan 2012) as well as lipid metabolism (Lee and Dong 2017), which further influence the TCA cycle and is a vital metabolite for germ cells (Jutte et al. 1981), despite its low abundance in seminal fluid (Evans and Setchell 1978). We thus detected whether disorders of insulin signaling pathway in *lushu-null* males influence those related pathways. As the results showed, several key genes located on these pathways including glucose metabolism, TCA cycle and lipometabolism displayed disrupted expression patterns (Fig. 5b, supplementary fig. 11, Supplementary Material online). We further quantified the insulin-like peptide (ILP) levels, a pivotal regulator of glucose homeostasis and lipid metabolism, through ELISA analysis. Results proved that ILP levels were significantly higher in *lushu-null* males than that in normal ones (Fig. 5c; Student *t*-test, *P* = 0.0000). By considering the disrupted expression profiles of genes associated with glucose metabolism, the TCA cycle, and lipid metabolism pathways, we propose that *lushu* is associated with energy metabolism pathways in *P. xylostella*.

### The *lushu* Gene is a Z Chromosome Linked Gene and Fixed in the Population

**Fig. 6.**
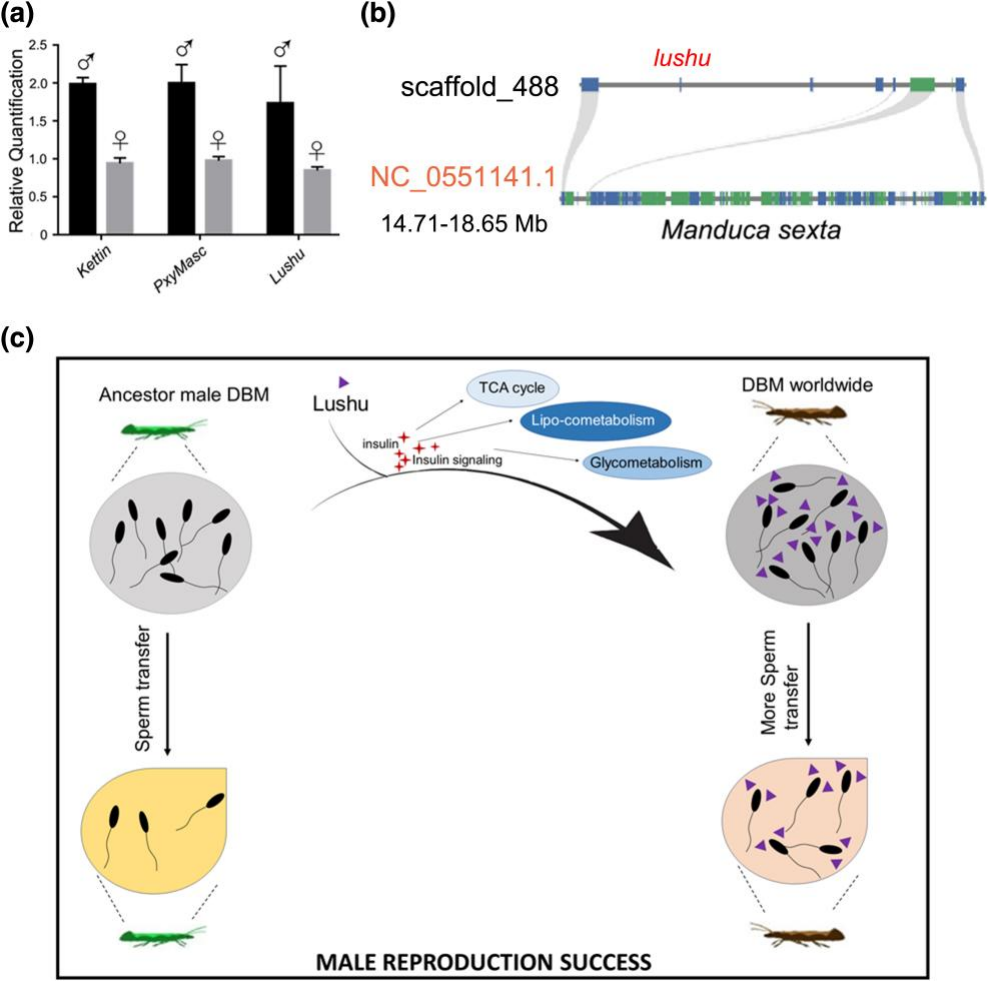
Evolutionary origination and model of DBM employing Lushu to achieve evolutionary success. a) *Lushu* is Z-linked in *P. xylostella*. b) Synteny analysis of *lushu* and its flanking genes. The *lushu* and its flanking genes have no obvious synteny blocks in *M. sexta*. c) Putative models for functions of *lushu* in *P. xylostella*.

To confirm that *lushu* is not a “PAV” (Presence and Absence Variation) event exclusive to certain individuals, we conducted a global assessment of its prevalence in various populations. Results showed that *lushu* existed in all detected populations collected worldwide (supplementary fig. 12a, Supplementary Material online), indicating that *lushu* has been fixed in the genome of *P. xylostella* as soon as it emerged. We also assessed the genomic copy number of the lushu gene, along with two Z-chromosome-linked marker genes (*Kettin* and *PxyMasc*). The three genes exhibit a consistent pattern with two copies in males and only one copy in females, suggesting that *lushu* was also a Z-linked gene (Fig. 6a). Further analysis involving synteny and homologous searches indicated that genes on scaffold_488, where *lushu* was located, did not exhibit clear synteny blocks in other species (Fig. 6b, supplementary fig. 12b, Supplementary Material online), despite a few neighboring genes having orthologs in other *Lepidoptera* species, such as *Bombyx mori*, *Danaus plexippus*, *Manduca sexta*, *Atethmia centrago*, and *Colias croceus* (Fig. 6b, supplementary fig. 12b, Supplementary Material online).

Based on the collective findings, our proposal is that *P. xylostella* possesses an orphan gene that codes a *P. xylostella* specific sperm protein, which improves male reproductive success by enhancing sperm competition (Fig. 6c). This gene is a contributing factor to high reproductive capability, functioning by affecting the energy metabolic related pathway in this species.

## Discussion

Orphan genes, which arise with new functions, are thought to play a major role in driving lineage-specific adaptive evolution (Kaessmann 2010; Wissler et al. 2013). However, the origination of orphan genes is still poorly understood. Previous studies have suggested several mechanisms responsible for formation of the orphan genes, including gene duplication, frame-shift mutations, de novo origination, horizontal gene transfer, and unexplained (unknown) origination (Wu et al. 2011; Wissler et al. 2013; Sun et al. 2015). In case of the orphan gene *lushu*, we inferred it as the unexplained origination since we were unable to determine its homologous sequences through similarity searches. Even though a small number of neighbor genes have orthologs in other *Lepidoptera* species, the ancestral sequence of *lushu* remains largely indistinguishable by similarity searches. In light of the *Lushu*'s presence in different populations worldwide, we believed that it is not a transient gene. Considering the presence of the *lushu* gene on the Z chromosome, which is significantly conserved among Lepidoptera (Fraisse et al. 2017), it is plausible that *lushu* may not be necessarily new but rather has undergone rapid evolution. This could lead to sequence divergence, which might pose challenges for identification, as previously described (Vakirlis et al. 2020; Weisman et al. 2020).

Orphan genes with functions associated with the adaptive success are more likely to be fixed and retained in genomes (Wissler et al. 2013). It is well known that genes associated with male reproductive function and fitness undergo rapid evolution through the origination of new ones (Swanson and Vacquier 2002; Haerty et al. 2007). The aim of this study was to investigate the mechanism behind the orphan gene, *lushu*, which facilitates male reproductive success in *P. xylostella*. Our findings reveal that Lushu is a sperm protein with high abundance. Additionally, Lushu deficiency resulted in low male reproductive success including a low male mating rate, early death, and poor sperm competition. In *D. melanogaster,* increased lifespan has been shown to enhance reproductive success when mating with virgin females in both competitive and noncompetitive conditions (Service 1993). These findings confirm that the introduction of genetic novelty to the sperm proteome occurs concurrently with the emergence of phenotypes related to male fitness advantage (Friedlander et al. 2005). All the evidences lead us to conclude that *lushu*, which originated as an orphan gene, became established in the genome of *P. xylostella* by acquiring beneficial functions, conferring advantages to the organism.

One of the main concerns is to determine whether Lushu is a sperm protein (SP) rather than a seminal fluid protein (SFP), given our detection of Lushu in the testes, seminal vesicles and male accessory glands (Fig. 1e, supplementary fig. S3c, Supplementary Material online). We speculated that Lushu is a sperm protein based on the following pieces of evidence. Firstly, we isolated sperm samples from male seminal vesicles and washed them as previously described (Whittington et al. 2015) before LC-MS/MS analysis. Secondly, prior research indicated that SFPs were primarily produced and located in the male accessory glands, and did not bind to sperm until mixing in the ejaculatory duct (Hurtado et al. 2022). However, we identified the localization of Lushu on the sperm bundles in the testes. Lastly, it was observed that the abundance of Lushu did not significantly decrease in the male accessory gland (MAG) after mating (NSAF = 0.00263 before mating *vs.* NSAF = 0.00256 after mating, Student's *t*-test, *P* = 0.11). In contrast, SFPs have been shown to become significantly less abundant in the MAGs or ejaculatory duct post-mating (Sepil et al. 2019; Hurtado et al. 2022; McCullough et al. 2022). We also observed that *lushu* is expressed at low levels in the testis compared to the male reproductive tract. This is not the first time that high expression of sperm proteins has been observed in nontestis tissues. Previous studies showed that many sperm structural proteins have expression in nontestes tissues, such as the male accessory gland (Degner et al. 2019; McCullough et al. 2022). Recent studies in *D. melanogaster* have demonstrated that certain SFPs can bind to sperm prior to mixing in the ejaculatory duct, contributing to various functions (Garlovsky et al. 2022; Hurtado et al. 2022; McCullough et al. 2022). McCullough et al. (2022) had specifically categorized these proteins as testis/reproductive gland proteins (testis/RG-proteins) rather than SFPs. Additionally, Garlovsky et al. (2022) reported the detection of such testis/RG-proteins by washing purified sperm with strong anionic detergent (Triton X-100), which disrupted plasma membranes. They also uncovered additional such proteins after washing with high molar salt, which is presumed to weaken ionic bonds and eliminate nonspecific protein binding to sperm (Garlovsky et al. 2022). Therefore, further investigations are warranted to explore the possibility of Lushu functioning as testis/RG-proteins that bound to sperm.

In male adults of *P. xylostella*, spermatogenic cells are organized into cysts, each of which generates sperm bundles, a characteristic mode of sperm aggregation. Multiple studies have revealed that sperm bundles perform crucial functions that can potentially enhance sperm transfer. These include supplying nutrients, safeguarding the acrosomes, and defending sperm against toxins secreted by rival males (Holman and Snook 2006; Takami and Sota 2007). In opossum (Moore 1996), fishflies (Hayashi 1998) and wood mice (Moore et al. 2002), aggregated sperm has been shown to enhance swimming speed, lending credence to the motility hypothesis (Ishijima et al. 1999). However, in *P. xylostella*, how *lushu* affects sperm transfer is still unclear and required further investigation. A reduction in the number of sperm transferred to females can lead to a decrease in sperm competition and egg production in offsprings (Bloch Qazi and Wolfner 2003; Avila and Wolfner 2017; Parker 2020; Laugen et al. 2022). Our results showed that when mated with mutant males once (30 min), the number of eggs in offspring decreases, sperm competitiveness weakens, but the hatching rate remains unchanged. These results suggested that the Lushu protein was not a core protein responsible for egg fertilization and development. Therefore, *lushu* may have impact on sperm accumulation, ultimately leading to the decreased fertility. This phenomenon has also been observed in various species, including *D. melanogaster* (Bloch Qazi and Wolfner 2003), *Struthio camelus* (Malecki and Martin 2003), *Neoseiulus womersleyi*, and *Neoseiulus longispinosus* (Ullah et al. 2017).

How these recently originated genes became integrated into pathways and how they conferred a male-related fitness advantage remain primarily unexplored (Yeh et al. 2012). We conducted a preliminary investigation into the pathways that *lushu* might be involved in. Notably, we found significantly elevated ILPs levels in *lushu-null* males, a critical regulator of glucose homeostasis and lipid metabolism in insects. These findings suggested that *lushu* may be related with pathways associated with energy metabolism to influence certain sperm traits such as ejaculation. Previous study reported that energy metabolism is a key factor that supports sperm functions (Miki 2007). For example, disorders of the insulin related pathway destabilize glucose homeostasis, which further affects the maintenance of spermatogenesis in vivo and the preservation of sperm fertility in male rats and humans (D’Cruz et al. 2012a, 2012b; Dias et al. 2014; Neirijnck et al. 2019). The seminal proteins insulin-like growth factor I (*IGF-IR*) correlated with semen quality and sperm motility in humans (Glander et al. 1996). And genes in the insulin superfamily involved in sperm motility and other functions have been observed in many species including humans (DaJusta et al. 2011), rodents (Filonzi et al. 2007), boars (Henricks et al. 1998), and domestic ruminants (Pitia et al. 2017). However, further investigations are needed to clarify the specific mechanisms linking *lushu* to energy metabolism. We must also consider the possibility that a lack of Lushu could alter the seminal ejaculate production or transfer, which thereby influencing the metabolic pathways. Previous studies in *Drosophila* highlighted the pivotal role of sex peptide (SP) in regulating the assembly of lipid microcarriers. Males lacking SP have shown disrupted transfer of multiple seminal proteins to females, potentially leading to systemic effects on metabolic processes (Wainwright et al. 2020). Similarly, in *D. melanogaster*, Acp36DE served as a testis/RG-proteins binding to sperms within the testis and seminal vesicles (Garlovsky et al. 2022; McCullough et al. 2022). Mutations in Acp36DE have been linked to slower sperm storage without evident changes in the uterine post-mating (Avila and Wolfner 2017).

The analysis of *lushu* expression profiles using qPCR revealed higher expression levels under conditions of low competition intensity and lower expression levels under high competition intensity. Consistent with previous study in *Drosophila* (Gallup et al. 2006), our results suggested that the content of sperm proteins plastically adjusted in relation to sperm competition intensity. One possible explanation for this observation is that males exposed to higher levels of competition have a greater quantity of sperm compared with males that are solitary. In *D. melanogaster*, it has been demonstrated that males have the ability to adjust their sperm production along a competition gradient, meaning that exposure to competition intensity can result in increased sperm production (Moatt et al. 2014; Hopkins et al. 2019). Several genes with similar expression profiles have been previously identified in fruit flies, but their functions remain unknown (Hopkins et al. 2019). In two gobiid fishes, the grass (*Zosterisessor ophiocephalus*) and black goby (*Gobius niger*), study also showed that the sperm expenditure was adjusted according with sperm competition. These sperm allocation strategies were expected to evolve if males were able to estimate the number of competitors and had the opportunity to allocate their sperm strategically (Pilastro et al. 2002).

Effective genetic management requires monitoring target genes that confer higher reproductive capacity. However, when dealing with closely related species, there is a risk of inadvertently affecting nontarget pests due to sequence similarities. To minimize this risk, identifying an orphan gene that is specific to the target species is essential. In this study, we discovered an orphan gene called *lushu* that regulates male reproductive success in *P. xylostella* through the energy metabolism pathway. This novel finding sheds light on the high reproductive capacity of *P. xylostella* males during fitness evolution and can guide genetic manipulation strategies for improved pest control.

## Materials and Methods

### Insects and Husbandry

The *G88* strain of *P. xylostella* was first collected by the New York State Agricultural Experiment Station and has been maintained on artificial diet without exposure to insecticides since 1988 (Shelton et al. 1991). It was established by Dr. Anthony M. Shelton (Cornell University, USA) and provided to the Institute of Applied Ecology, Fujian Agriculture and Forestry University, in 2016. Since then, the *G88* strain has been maintained in our lab at 26 ± 1 °C and 65% RH with a photoperiod of 16:8 h (L:D). A 10% honey solution was provided as food to the adults for further egg laying.

### Sperm Proteome

About 180 *G88* males were anesthetized at 1 d post-eclosion and dissected to remove the seminal vesicles from the abdomens (supplementary fig. S4a, Supplementary Material online). Fluid samples were collected in three replicates by carefully puncturing the seminal vesicle using acupuncture needles. Proteins were extracted according to procedures described in Whittington et al. (2015). Samples of 2 μg of total proteins were prepared and separated by 10% SDS-PAGE (supplementary fig. S4b, Supplementary Material online). Then 30-μg samples of total proteins were sent to Applied Protein Technology (Shanghai, China) for LC-MS/MS analysis. We used Proteome Discoverer 1.3 to convert the obtained data and Mascot v2.3.02 to query predicted proteins in the DBM database (You et al. 2013) to identify the sperm proteins. Relative protein abundance was assessed by calculating the normalized spectral abundance factor (NSAF) (http://doi.org/10.1002/rcm.7829) as described by Kaysheva et al. (Bubis et al. 2017; Kaysheva et al. 2018).

### Proteins that Getting into Females

About 200 *G88* females were anesthetized within 30 min after mating with *G88* males and dissected to remove the bursa (supplementary fig. S4c, Supplementary Material online). The proteins were collected and extracted according to procedures described before (supplementary fig. S4d, Supplementary Material online) (McCullough et al. 2022) using LC-MS/MS analysis. Then the proteomics data of the proteins that getting into female bursa were obtained using the same method described above (see the method of sperm proteome).

### Characterization of the Orphan Genes

To identify orphan genes that were evolved specifically in *P. xylostella*, the predicted proteins in the nr (nonredundant) database were compared as described in Sun et al. (2015) for *B. mori* and Domazet-Loso and Tautz (2003) for *D. melanogaster*. Predicted protein sequences in *P. xylostella* were collected from China National Center for Bioinformation (https://www.cncb.ac.cn/) with accession number GWHERDJ00000000. Firstly, all predicted proteins of *P. xylostella* were utilized to query the predicted proteins of six other *Lepidopteran* insects retrieved from https://www.ncbi.nlm.nih.gov/, including *B. mori* (accession: GCA_030269925.2), *Heliconius melpomene* (accession: GCA_000313835.2)*, M. sexta* (accession: GCA_014839805.1)*, D. plexippus* (accession: GCA_018135715.1), *C. croceus* (accession: GCA_905220415.1), and *Spodoptera litura* (accession: GCA_002706865.3) via BLASTP with an E-value threshold > 10-3. The remaining sequences were then used to query lepidopteran EST sequences using tBLASTN at http://www.cdfd.org.in/wildsilkbase/, http://butterflybase.ice.mpg.de/and http://bioweb.ensam.inra.fr/spodobase/ excluding all *P. xylostella* EST sequences. The proteins of *P. xylostella* with homologous matches in other species were also discarded from further analysis. The remaining proteins were then used as BLASTP queries to search the nr database. Finally, the remaining set of proteins corresponded to orphan genes identified in *P. xylostella* that had no homology to genes in other species.

We also investigated the emergence of *P. xylostella* orphan genes according to previous studies (Wu et al. 2011; Wissler et al. 2013; Sun et al. 2015). Paralogs of the *P. xylostella* orphan genes were inferred using BLASTP searches against all *P. xylostella* proteins with the cutoff E value was 0.001. The result hit sequences were defined as gene duplications. Overlapping with transposable elements (TEs) was identified based on CDS overlaps of DBM orphan genes with TE coordinates using BLASTN searches with E-value cutoff of 10-5. Overlapping gene models were inferred as pairs of genes whose CDS overlaps by at least 30 nt on opposite strands, which was described before (Wissler et al. 2013). For the de novo origination, we used the pipeline performed before in human genome (Wu et al. 2011). All the orphan genes were searched using BLAT against 6 other *Lepidoptera* genomes, including *B. mori*, *H. melpomene*, *M. sexta*, *D. plexippus*, *C. croceus*, and *S. litura* to identify the orthologous regions. These orthologous sequences that contained a frame-shift or premature stop codon that prevented the translation of a protein of at least 80% of the size in DBM were considered to be nonprotein coding. That is, to be a candidate de novo originated orphan gene, in addition to coding a protein in *P. xylostella* genome, the gene must have been present, but disrupted (i.e. noncoding regions) in other *Lepidoptera* genomes. Orphan genes, which cannot be classified by the origin models reported before, were considered as unknown origin genes.

We then characterized the GC contents, exon numbers, sequence lengths, and isoelectric points of the orphan genes from *P. xylostella* and compared each of these parameters with those of the conserved genes in *P. xylostella*. Isoelectric points were calculated using DAMBE (Xia 2013). Further, we wrote scripts to calculate the GC contents, exon numbers, and sequence lengths of the orphan genes we identified in *P. xylostella*.

### Expression Profiling of the Orphan Genes

Samples of eggs, first instar larvae, second instar larvae, third instar larvae, forth instar males, forth instar females, female pupae, male pupae, female adults, and male adults of *G88* were previously collected (He et al. 2012). The Illumina HiSeq 2500 platform was used to generate paired-end reads to calculate the transcript abundances of genes in different genders and developmental stages as described before (He et al. 2012; You et al. 2013). We profiled the expression patterns of the orphan genes based on these RNA-Seq data.

### Cloning of *Lushu* and Analysis of its Transcript Expression

*G88* male adults were collected (*n* = 10) and total RNA was extracted using an Eastep Super Total RNA Extraction Kit (Promega, Shanghai, China). cDNA was synthesized using the FastKing gDNA Dispelling RT SuperMix (TIANGEN, Beijing, China) according to the manufacturer's protocols. The full-length *lushu* gene was amplified using PCR in 25 μl reactions (12.5 μl of 2 × Phanta Max Buffer (Vazyme Biotech, Nanjing, China), 1 μl cDNA template, 2 μl primers (supplementary table S9, Supplementary Material online), 0.5 μl Phanta Max Super-Fidelity DNA Polymerase, 0.5 μl dNTP Mix, and 8.5 μl ddH_2_O). The thermal cycling program for PCR was set as follows: 95 °C for 3 min, followed by 34 cycles of denaturation at 95 °C for 30 s, annealing at 59 °C for 30 s, and extension at 72 °C for 2 min. Results were examined by agarose gel electrophoresis and the amplified products were further purified using a Gel Extraction Kit (Omega, Morgan Hill, GA, USA). We cloned the products into the Pjet1.2/blunt Cloning Vector using the CloneJET PCR Cloning Kit (Thermo Scientific, Waltham, MA, USA) and sent clones to Biosune Biotech Company (Fuzhou, China) for DNA sequencing. During amino acid sequence analysis, signal peptides were predicted using the SignalP Server 5.0 (http://www/cbs.dtu.dk/services/SignalP/). qPCR was performed to validate expression of *lushu* using primers listed in supplementary table S9, Supplementary Material online. For qPCR reactions, the master mix (GoTaq qPCR Master, Promega, Madison, USA) contained 10 μl qPCR Master Mix, 7.2 μl ddH_2_O, 2 μl cDNA, and 0.8 μl primer. The qPCR program was set as follows: 95 °C for 10 min followed by 44 cycles of 95 °C for 5 s, 60 °C for 30 s, and 72 °C for 60 s, followed by a final extension period of 72 °C for 5 min. Relative quantification was performed using the comparative 2^−ΔΔ^*^C^*_T_ method. We assessed expression of the gene Rlk/Itk-binding protein (*RIBP)* as the control gene for qRT-PCR in our study. The samples were collected at different developmental stages including egg, larva (male and female samples were mixed in equal amounts), pupa (first- to forth-day male pupa was prepared separately), female adult, and male adult (first- to fifth-day male adults were collected separately). Moreover, various tissues of male adults, including head, thorax, testis, and testis remnant (containing accessory gland, vas deferens, and seminal vesicles), were collected separately. Four replicates were prepared for each sample.

### Immunofluorescence Assays

The reproductive system of 1-d-old adult males including testis and reproductive tract were fixed at 4 °C overnight in 4% paraformaldehyde (Solarbio, Beijing, China). Sperm samples were obtained by delicately dissecting the testis from 1-d-old moth and then fixed in 4% paraformaldehyde at room temperature for 30 min (Solarbio, Beijing, China). These samples were then washed with PBS (pH 7.2 to 7.4) and the samples were incubated with 0.1% Triton X-100 (Solarbio, Beijing, China) at room temperature (RT) for 4 h. Samples were washed again with 1× TBST and incubated with 3% blocking buffer (Yeasen Biotech Co. Ltd, Shanghai, China) at RT for 1 h. After washing again with 1× TBST, the samples were incubated with purified rabbit anti-Lushu antiserum (dilution 1: 200) at 4 °C overnight, and then incubated with HRP-Goat Anti-Rabbit IgG (dilution: 1:200) (Boster, Wuhan, China) at RT for 2 h in the dark. These samples were placed on glass slides, covered with a drop of glycerin, and then gently covered with coverslips. Samples were observed using a Leica SP5 confocal laser-scanning microscope (Leica, Wetzlar, Germany). Samples included *G88* males, *lushu-null* males, and silk gland control from *G88* larvae.

### DNA Extraction and Genomic Copy Number Estimation of *Lush*

DNA of Male and female *P. xylostella* pupae was extracted using TIANcombi DNA Lyse&Det PCR Kit (TIANGEN, Beijing, China). Extracted DNA samples were quantified using a NanoDrop 2000 Spectrophotometer (Thermo Fisher Scientific).

Previously proved Z-linked genes, including *Pxy-Masc* and *kettin*, were used as the positive control in our study with primers listed in supplementary table S9, Supplementary Material online. *Defensin*, a known autosomal gene, was used as a reference gene using the published primers Def-qF: CCAACCGGTCAACAGTCAAAATG and Def-qR: TCTCGGGTAACACAAAGCACTCG (Harvey-Samuel et al. 2020). For the qPCR reaction, the master mix (*PerfectStart* Green qPCR SuperMix, TRANSGEN, Beijing, China) contained 10 μl 2*×PerfectStart* Green qPCR SuperMix, 7.2 μl ddH2O, 2 μl cDNA, and 0.8 μl primer. The qPCR program was set as follows: 94 °C for 30 s followed by 44 cycles of 94 °C for 5 s, 60 °C for 30 s. Melt curve was set as follows: from 60 °C to 95 °C, increasing 0.5 °C every 5 s. Relative quantification was calculated using the 2^−ΔΔ^*^C^*_T_ method to estimate genomic copy numbers of *lushu* for males and females. We totally prepared four biological replicates.

### Preparation of sgRNA and Cas9

The target site for the sgRNA containing 5′N20NGG-3′ (the PAM sequence is underlined) was chosen within the *lushu* exon in *P. xylostella* (there are no introns in the*lushu* gene) (Fig. 2a). The transcription template for in vivo sgRNA synthesis includes two oligonucleotides. The first contains the T7 polymerase binding site and the target sequence (5′-TAATACGACTCACTATA**GGCGTGTTCAGAGGCGCTCC**GTTTTAGAGCTAGAAATAGCAAGTTAAAATAAGGCTAGTCC-3′) and the second one contains the common sgRNA sequence (5′-AGCACCGACTCGGTGCCACTTTTTCAAGTTGATAACGGACTAGCCTTATTTTAACTTGCTATTTCTAGCT-3′). The sgRNA target sequence for the *lushu* gene was listed in supplementary table S10, Supplementary Material online. The 50 μl reaction used to amplify the sgRNAs by PCR contained 10 μl 5 × PrimeSTAR Buffer, 10 μl 2.5 mM dNTPs, 4 μl primers, 0.5 μl PrimeSTAR HS DNA Polymerase, and 25.5 μl ddH2O (PrimeSTAR HS DNA Polymerase, Takara), and was run with the following program: 98 °C for 2 min, 20 cycles at 98 °C for 10 s, and 72 °C for 20 s. PCR products were examined by agarose gel electrophoresis and purified using a Gel Extraction Kit (Omega). We used the HiScribe T7 Quick High Yield RNA Synthesis Kit (New England Biolabs, Ipswich, MA, USA) to generate the sgRNAs in vitro and purified them using phenol-chloroform extraction and ethanol precipitation as described in Liu et al. (2020). The amplified sgRNAs were then stored at −80 °C for further use.

Using a linearized PTD1-T7-Cas9 vector (Huang et al. 2016) as template, we synthesized the Cas9 mRNA in vitro using a HiScribe T7 Quick High Yield RNA Synthesis Kit (with tailing) (New England Biolabs). The Cas9 mRNA was purified using the same method as described for purification of the sgRNA. The Cas9 protein (GenCrispr Cas9-N-NLS Nuclease) was purchased from GeneScript Corporation (Nanjing, China)

### Embryo Microinjection for Targeted Mutagenesis of *Lushu*

A parafilm sheet (2 cm × 5 cm) precoated with dry radish seedling powders was provided for *G88* females to lay eggs on and was collected within 10 min and affixed to a glass slide for injection of embryos with a mixture containing Cas9 mRNA (300 ng/μl) and sgRNA (100 ng/μl). Injections were performed using an IM 300 Microinjector (Narishige, Tokyo, Japan) mounted on an SZX16 Stereo Microscope (Olympus, Tokyo, Japan) and finished within 90 min of egg laying. Injected eggs were incubated without light at 26 ± 1 °C in Petri dishes containing moist tissue paper. The moist tissue paper was removed after hatching and the hatched first instar larvae, representing G_0_, were raised on an artificial diet to estimate the hatchability injected eggs and the survival rate of G_0_ (supplementary table S11, Supplementary Material online).

### Homozygous Mutant Strain Screening and Establishment

The surviving adults of G_0_ were mated with virgin *G88* adults in single-pair matings (supplementary fig. 13, Supplementary Material online). The G_0_ pairs were collected for subsequent genomic DNA isolation after oviposition. We amplified the region spanning the sgRNA target site from genomic DNA using PCR and retained offspring (G_1_) of G_0_ pairs containing mutations. G_1_ siblings were mated in single pairs to produce G_2_ offspring, and after we genotyped the G_2_ offspring with heterozygous parents, only those that shared the same allelic mutations were retained. G_2_ siblings were single-pair mated to produce G_3_ progeny and only G_3_ progeny with homozygous mutant parents were kept to build the homozygous mutant strains. In this study, we performed another mating between a pair of siblings to confirm the homozygous mutant parents.

Genomic DNA was isolated from whole adults after oviposition using Tissue DNA Kit (TIANGEN, Beijing, China) to screen for CRISPR/Cas9-mediated mutations. Amplicons spanning the sgRNA target sites for the *Lushu* gene were generated using primers listed in supplementary table S12, Supplementary Material online and the PCR program described in the section above describing the cloning and expression analysis of *Lushu*. PCR products were sent for sequencing to Biosune Biotech Company.

### Validation of Lushu Deletion

To further verify the knockout of Lushu, 2 μg of total proteins from the male adult abdomen of *G88* and mutant strains (from three replicates for each strain) were prepared and separated using 10% SDS-PAGE. Gel sections containing proteins with molecular weights ranging from ∼15 to 35 kDa were excised and sent to Applied Protein Technology (Shanghai, China) for LC-MS/MS analysis as described above in the section describing identification of sperm proteins in *P. xylostella*.

### Genome-wide Prediction CRISPR Off-target Mutations

We identified CRISPR off-target mutations through whole-genome sequencing (WGS). Statistics of the WGS data was listed in supplementary table S13, Supplementary Material online. Genomic DNA was extracted from 1-day-old male adults using the TIANamp Genomics DNA Kit (TIANGEN). For each sample, at least 500 ng of DNA was utilized to construct the sequencing library following the MGIEasy Universal DNA Library Preparation Kit (Shenzhen, China). The final libraries were sequenced using DNBSEQ-T7 (paired-end 150-bp). The reference genome and its annotation for *P. xylostella* were downloaded from the China National Center for Bioinformation (Accession number: GWHERDJ00000000; https://www.cncb.ac.cn/). Initially, raw reads underwent filtering with Trimmomatic (Version 0.36, MINLEN:75) (Bolger et al. 2014). Clean reads were then mapped to the reference genome using BWA (Version 0.7.17) with default parameters (Li and Durbin 2009). SNPs were called with BCFtools (Version 1.19) (Li 2011) and Genome Analysis Toolkit (GATK, Version 3.8, -stand_call_conf 30.0) (Mckenna et al. 2010). For indels, BAM files were realigned using the GATK (parameters: -T RealignerTargetCreator, IndelRealigner) to obtain accurate indels. To ensure high-quality SNPs and indels, common variations detected by GATK and BCFtools were merged (-T Selectvariations). SNPs and indels were filtered with the following parameters: QD < 20.0 || ReadPosRankSum <8.0 || FS >10.0 || QUAL < $MEANQUAL).

The off-target prediction pipeline was previously described (Li et al. 2019) and is illustrated in supplementary fig. 14, Supplementary Material online. The sgRNA was aligned against reference genome using Cas-OFFinder algorithm with up five mismatched as described (Bae et al. 2014; Li et al. 2019) to predict the candidate CRISPR-edited variations. The flanking 20-bp of the candidate edited sites were used to search for corresponding variations in *lushu-null* males to generate a likely off-target mutation. Since the stain used for the reference is not *G88*, likely off-target mutations existing in both *G88* and *lushu-null* males were excluded from further analysis. Finally, the final off-target mutations were visualized in reference, *G88*, and *lushu-null* using the IGV tool to confirm the Cas9-edited mutations.

### Male Longevity and Offspring Numbers

Five replicates (each containing 30 male individuals) of G88 and *lushu-null* strains were used to record timelines for life table analysis. For fecundity analysis, males and females were kept separately from the 4th pupae until eclosion. Because newly eclosed DBM moths are not active for mating for the first 12 h, we isolated them separately in plastic microfuge tubes for 18 h before mating to virgin G88 females in a plastic box. Fifteen newly eclosed males (18-hour-old) and females (18-hour-old) (*G88*♀ × *G88*♂ compared to *G88*♀ × *lushu-null*♂) were used to perform one-to-one mating. Each mating pair was placed in one plastic box. Each plastic box contains a parafilm sheet (2 cm × 3 cm) precoated with dry radish seedling powder for egg laying. A 10% honey solution was provided as nutrition of adults. The parafilm sheet was changed every 24 h, and the egg numbers and hatchability rates were calculated. Significance was analyzed using Student's *t*-test. To be noticed, these data on egg-laying capacity is based on multiple matings for both males and females.

Simultaneously, we monitored the daily egg-laying capacity of *G88* females after mating once (30 min) with *G88* and *lushu-null* males. Males and females were housed separately from the forth pupal stage until eclosion. Male adults were individually isolated in plastic microcentrifuge tubes for 18 h prior to mating with virgin *G88* females in a plastic box. Twenty newly eclosed males (18-hour-old) and females (18-hour-old) (*G88*♀ × *G88*♂ compared to *G88*♀ × *lushu-null*♂) were used to perform one-to-one mating. Each mating pair was placed in one plastic box. Each plastic box contains a parafilm sheet (2 cm × 3 cm) precoated with dry radish seedling powder for egg laying. A 10% honey solution was provided as nutrition of adults. We separated males and females after mating once (30 min) and recorded the daily egg-laying capacity of the females. The parafilm sheet was changed every 24 h, and the egg numbers. Significance was analyzed using Student's *t*-test.

### Mating Rate Analysis

The male mating rate was estimated for *G88* and *lushu-null* individuals. We conducted a comparison of the mating rates and preparation times for mating between *G88* males and *lushu-null* males. Matings lasting more than 30 min were considered as effective mating, and the time required for effective mating was recorded. Males and females were kept separately from the forth pupal stage until eclosion. Then we isolated them separately in plastic microfuge tubes for 1 d before mating in a plastic box. A total of 10 biological replicates were prepared. Each replicate contained six pairs, with each pair consisting of one *G88* male (18-hour-old) and one virgin *G88* female (18-hour-old) placed in separate plastic boxes with a 10% honey solution provided as nutrition. We recorded mating status every 30 min. The mating rate of the sexually mature *G88* adults can reach over 50% within 30 min, with the majority of adults able to compete mating within 2 h, consistent with previous study (Zou et al. 2022). Thus, we observed for a total of 3 h. The significance of the obtained results was assessed using Student's *t*-test.

### Testis Size

The testis was dissected from the 1-day-old virgin male after eclosion and measured with an ultra-depth three-dimensional microscope (Keyence, China) operated by software (VHX, Beijing, China). We prepared 15 males as replicates for each *P. xylostella* strain. The differences of testis size were statistically assessed using Student's *t*-test.

### Sperm Count and Sperm Vitality

Adult males were anesthetized at the time of 1-d after eclosion and dissected to collect the seminal vesicles and testis from abdomens. The fluid from the seminal vesicles was collected on a glass slide by carefully puncturing them using acupuncture needles and stained with DAPI incubating for 15 min. Similarly, seminal fluid from the testes was collected on a glass slide by gently tearing it apart using tweezers and stained with DAPI incubating for 15 min. The sperm bundles quantities were counted and photographed before and after mating using a Leica SP5 confocal laser-scanning microscope (Leica, Wetzlar, Germany). Totally, 100 male adults were used in this analysis (25 adults from each line). Significance was analyzed using Student's *t*-test. Sperm bundles retention ratio was calculated as the number of sperm bundles in the testes after mating divided by the number of sperm bundles in the testes before mating:

Sperm bundles retention ratio = $\frac{N_{\mathrm{sperm}\;\mathrm{bundle}\;\mathrm{numbers}\;\mathrm{after}\;\mathrm{mating}}}{N_{\mathrm{sperm}\;\mathrm{bundle}\;\mathrm{numbers}\;\mathrm{before}\;\mathrm{mating}}}$; Significance was analyzed using Student's *t*-test.

To assess the vitality of sperm cells, we quantified the number of dead sperm cells. Following the protocol of the Calcein-AM/PI Double Stain Kit (Yeasen, Shanghai, China), we stained dead sperm cells in the seminal vesicles with red dye, while live cells were stained with green dye.

To collect the bursa samples, we conducted one-on-one mating between 1-d-old males and 1-d-old females (one pair in a separate box). The females were then collected and flash frozen in liquid nitrogen 30 min after mating. Fluid from the bursa was collected on a glass slide by gently tearing it apart using tweezers and stained with DAPI incubating for 15 min. The number of sperm cells were counted and photographed with Leica SP5 confocal laser-scanning microscope (Leica, Wetzlar, Germany). A total of 200 female adults were utilized, with 50 females mating with each line.

### Sperm Competition Assay

Two kinds of sperm-displacement assays, described here as offense and defense experiments, were performed following procedures previously described by Yeh et al. (2012). The offense experiment was carried out by effective mating of virgin *G88* females with virgin G88 males for 12 h. Next, each experimental female was transferred into a different plastic box to oviposit for 8 h (card 1 in Fig. 3). Then the experimental female moth was transferred to a new plastic box with a virgin *lushu-null* male to mate for 12 h. Females were next removed to another plastic box to oviposit for 12 h (card 2) and then transferred to a new plastic box to oviposit for another 24 h (card 3). After that (after 68 h), females were moved to the last box to oviposit until died before being discarded (card 4). For the defense experiment, we followed similar procedures except that the *G88* females were mated to *lushu-null* virgin males first and to *G88* virgin males last. For each experiment, card 1 was examined for the presence of hatched larvae to confirm effective mating. Females that were unsuccessful at the first mating or that died during the experiments were disregarded for further analysis. The number of eggs laid on card 2 and card 3, which reflects the actual competition between sperm from G88 and *lushu-null* males, was recorded and the eggs were genotyped. For each female, we calculated a sperm competition index depending on the genotypes of their offspring. The proportion of progeny from mating with the last male (known as P2), was calculated as:


$$P2_{\left(\mathrm{offense}\right)}=\frac{N(+/lushu)}{N(+/lushu)+N(+/+)}$$

and


$$P2_{\left(\mathrm{defense}\right)}=\frac{N(+/+)}{N(+/lushu)+N(+/+)}$$

During the experiment, a 10% honey solution was provided as adult nutrition. This assay was repeated three times. Differences in sperm competition among experiments were statistically assessed using Student's *t*-test and Fisher's Exact Test.

### Response of *lushu* Expression to Different Male Competition Intensity

Previous study in *Drosophila* explored a range of competitive scenarios that males experience, with individually housed males experiencing no competition, pairs experiencing low competition, and groups of eight experiencing high competition (Hopkins et al. 2019). Experimental males were identified at the forth instar stage and maintained individually at the pupa stage. We collected the males as virgins after eclosion and maintained them either in one individual male (no competition), in a pair of two males (low competition), or in a group of eight males (high competition) in separate plastic boxes (3 × 3 × 3 cm) for 12 h, 24 h, or 36 h. A 10% honey solution was supplied as food for the experimental males. Relative expression level of *lushu* was then examined using qRT-PCR separately for each grouping of one individual male, a pair of two males, and a group of eight males (Fig. 3b). Four replicates were prepared for this analysis. Relative quantification was calculated based on the 2^−ΔΔ^*^C^*_T_ method, using *RIBP* as the control gene. Significance was analyzed using Student's *t*-test.

### RNA Sequencing and GO Analysis

*G88* males and *lushu-8* males were collected at the time of one day after eclosion. Total RNA was extracted using an Eastep Super Total RNA Extraction Kit (Promega, Shanghai, China) following the manufacturer's instructions. RNAs extracted from experimental samples (three biological replicates) were sequenced on an Illumina HiSeq X Ten platform, yielding 29.9 to 35.3 million reads for each sample (supplementary table S14, Supplementary Material online). Reads trimmed using Trimmomatic (Bolger et al. 2014) were mapped against annotated DBM gene models using Bowtie2 (https://bowtie-bio.sourceforge.net/bowtie2/index.shtml) (Langmead and Salzberg 2012), and only the best alignments were retained. FPKM (fragments per kilobase of exon per million fragments mapped) were calculated using RSEM (Li and Dewey 2011) in the Trinity package (Haas et al. 2013). Differentially expressed genes (DEGs) were identified using DESeq2 (Love et al. 2014). Significantly differentially expressed genes were detected with a cutoff (*P* < 0.05 and |log_2_^change fold^| > 1.5).

GO term and KEGG pathway enrichments were analyzed using OmicShare tools (https://www.omicshare.com/tools/Home/Soft/getsoft). To further confirm the expression of genes that are potentially involved in pathways related to *lushu*, we performed qRT-PCR (primers are listed in supplementary table S15, Supplementary Material online) to analyze the potential target pathways. Expression level was analyzed using the delta-Ct method. The ΔC_T_ was calculated based on previous study (Zhao et al. 2014). Relative quantification was performed using the comparative 2^−Δ^*^C^*_T_ method. We assessed expression of the gene *RIBP* as the control gene for qRT-PCR in our study. Four replicates were prepared, with each replicate consisting of 10 male adults.

### ILPs Concentration Assay Using ELISA

Adult males of G88 and *lushu-null* were collected at 24 h after eclosion. The PBS (PH 7.4) was added to the samples and homogenized by grinders. Then we performed the centrifugation for 20 min at the speed of 3,000 r.p.m to collect the supernatant. Four replicates were set in this assay.

The ILPs levels of the samples were evaluated by ELISA assay (Insect Insulin (INS) ELISA kit, mlbio, Shanghai, China). This Kit assays the insect ILP concentrations using purified insect insulin antibody to coat microtiter plat wells. Adding the insect samples to the wells, the HRP labeled antibody combined with insulin to construct the antibody-antigen-enzyme-antibody complex. Then the TMB substrate solution was added to observe the color development reaction (changing blue) and the reaction was terminated by adding sulfuric acid solution. Color change was measured spectrophotometrically at wavelength of 450 nm. The concentration of ILPs is determined by comparing the O.D. of the samples to the standard curve. In this ELISA analysis, we conducted six biological replicates, with each replicate containing 10 adult males. Means were analyzed using Student's *t*-test. Standard curve of the ELISA assay to evaluate the ILP content is listed in supplementary fig. S15, Supplementary Material online.

### Synteny Analysis

Genome sequences of *B. mori* (accession: GCA_030269925.2), *H. melpomene* (accession: GCA_000313835.2), *M. sexta* (accession: GCA_014839805.1), *D. plexippus* (accession: GCA_018135715.1), *A. centrago* (GCA_905333075.3), and *C. croceus* (accession: GCA_905220415.1) were download from NCBI (https://www.ncbi.nlm.nih.gov/). To identify syntenic gene blocks between DBM and other *Lepidoptera* organisms, all-against-all BLASTP (E-value < 10^−5^) was performed for the genes of each genome pair. The synteny blocks were defined based on the presence of at least 1 synteny gene pairs using JCVI MCScan (https://github.com/tanghaibao/jcvi/wiki/MCscan-(Python-version) with the settings –dist = 1000 and -n 1.

Lushu • 《from The Classic of Mountains and Seas (山海经; *Shan Hai Jing*) • Nanshan Jing (南山经) Classic of the Mountains: South》

According to an ancient Chinese legend, a wild animal known as Lushu lived in the Niuyang mountains located in southwestern China long ago. Lushu was said to resemble a horse with a snow-white head and its body decorated in the patterns of a tiger with a red tail. The legend described the sounds Lushu made as sounding like people singing songs around the mountain. As the legend said, people longed to possess Lushu in hopes of plenty of children to show the prosperity of their family.

## Supplementary Material

msae142_Supplementary_Data

## Data Availability

No datasets were generated in the current study.
