# Supplementary material for: An Orphan Gene Enhances Male Reproductive Success in Plutella xylostella
Source: Mol Biol Evol. 2024 Jul 11;41(7):msae142. doi: 10.1093/molbev/msae142 (PMC11290247; doi:10.1093/molbev/msae142)
Supplement: msae142_Supplementary_Data [file msae142_supplementary_data.zip › Supplemental information.pdf]

# Supplemental information

## Orphan genes of *P. xylostella*

Among the 1100 orphan genes we identified in the *P. xylostella* genome, 242 had arisen by gene duplication, a major mechanism by which new genes originate (Yang, et al. 2013) in many organisms (Toll-Riera, et al. 2009; Donoghue, et al. 2011; Yang, et al. 2013). Further, we also identified 22 new genes containing TE-like sequences and five orphan genes that arose *de novo* (Supplementary Fig. 1a). As indicated by previous studies, *de novo* origination of new genes is rare (Levine, et al. 2006).

Compared with genes that are conserved across arthropod species, the orphan genes in *P. xylostella* differ substantially in that they exhibit shorter gene lengths, shorter protein lengths, fewer exons, higher GC contents, and higher isoelectric points (Mann-Whitney *U* test, $P$ <0.0001) (Supplementary Fig. 1b). Shorter gene and protein lengths and fewer exons have also been observed in newly evolved genes in other organisms including primates (Wu, et al. 2011), plants (Donoghue, et al. 2011), and other insects (Sun, et al. 2015), indicating that these features are common among new genes in eukaryotic organisms. However, differences in the GC contents of new genes compared to conserved ones are more complicated. The GC contents of new genes are significantly higher in some insect species such as *P. xylostella, Bombyx mori* (silkworm), and *Danaus plexippus* (monarch butterfly), and in the plant family Poaceae (Campbell, et al. 2007; Sun, et al. 2015) but are lower in species such as the butterfly *Heliconius melpomene*, the fruit fly *Drosophila melanogaster*, and the zebrafish *Danio rerio* (Domazet-Loso and Tautz 2003; Yang, et al. 2013). Furthermore, although the GC contents of new genes are unusual in most cases, the GC contents of new genes in some organisms including *Manduca sexta* do not significantly differ from those of conserved genes (Sun, et al. 2015). The reasons for differences among species in GC contents of new genes are still not known but might be related to the specific functions of GC-rich regions in each species. The theoretical isoelectric point (pI) of a protein encoded by a new gene might be related to its subcellular localization, solubility, and interactions (Nandi, et al. 2005; Kiraga, et al. 2007). Shifts in pI can affect protein

function (Khaldi and Shields 2011), which might contribute to adaptation to various environments (Nandi, et al. 2005). Therefore, the elevated pI of proteins encoded by the new genes that we observed in DBM might reflect newly evolved functions that could be relevant to DBM-specific adaptions.

## Reference


Campbell MA, Zhu W, Jiang N, Lin H, Ouyang S, Childs KL, Haas BJ, Hamilton JP, Buell CR. 2007. Identification and characterization of lineage-specific genes within the Poaceae. Plant Physiol 145:1311-1322.

Domazet-Loso T, Tautz D. 2003. An evolutionary analysis of orphan genes in Drosophila. Genome Res 13:2213-2219.

Donoghue MT, Keshavaiah C, Swamidatta SH, Spillane C. 2011. Evolutionary origins of Brassicaceae specific genes in Arabidopsis thaliana. BMC Evol Biol 11:47.

Khaldi N, Shields DC. 2011. Shift in the isoelectric-point of milk proteins as a consequence of adaptive divergence between the milks of mammalian species. Biol Direct 6:40.

Kiraga J, Mackiewicz P, Mackiewicz D, Kowalczuk M, Biecek P, Polak N, Smolarczyk K, Dudek MR, Cebrat S. 2007. The relationships between the isoelectric point and: length of proteins, taxonomy and ecology of organisms. BMC Genomics 8:163.

Levine MT, Jones CD, Kern AD, Lindfors HA, Begun DJ. 2006. Novel genes derived from noncoding DNA in Drosophila melanogaster are frequently X-linked and exhibit testis-biased expression. Proc Natl Acad Sci U S A 103:9935-9939.

Nandi S, Mehra N, Lynn AM, Bhattacharya A. 2005. Comparison of theoretical proteomes: identification of COGs with conserved and variable pI within the multimodal pI distribution. BMC Genomics 6:116.

Sun W, Zhao XW, Zhang Z. 2015. Identification and evolution of the orphan genes in the domestic silkworm, Bombyx mori. FEBS Lett 589:2731-2738.

Toll-Riera M, Bosch N, Bellora N, Castelo R, Armengol L, Estivill X, Alba MM. 2009. Origin of primate orphan genes: a comparative genomics approach. Mol Biol Evol 26:603-612.

Wu DD, Irwin DM, Zhang YP. 2011. De novo origin of human protein-coding genes. PLoS Genet 7:e1002379.

Yang L, Zou M, Fu B, He S. 2013. Genome-wide identification, characterization, and expression analysis of lineage-specific genes within zebrafish. BMC Genomics 14:65.

**Video S1: This video shows the mating behaviors differences between *G88* and *lushu-null*. The video showed a significant decrease in the mating rate for mutant males.**

## Supplementary Figure legends

**Supplementary figure 1.** (a) Identification of orphan genes in *P. xylostella.* (b) Characteristics of orphan genes comparing to the non-orphan genes. PxOGs: *P. xylostella* orphan genes; PxNOGs: *P. xylostella* non-orphan genes.

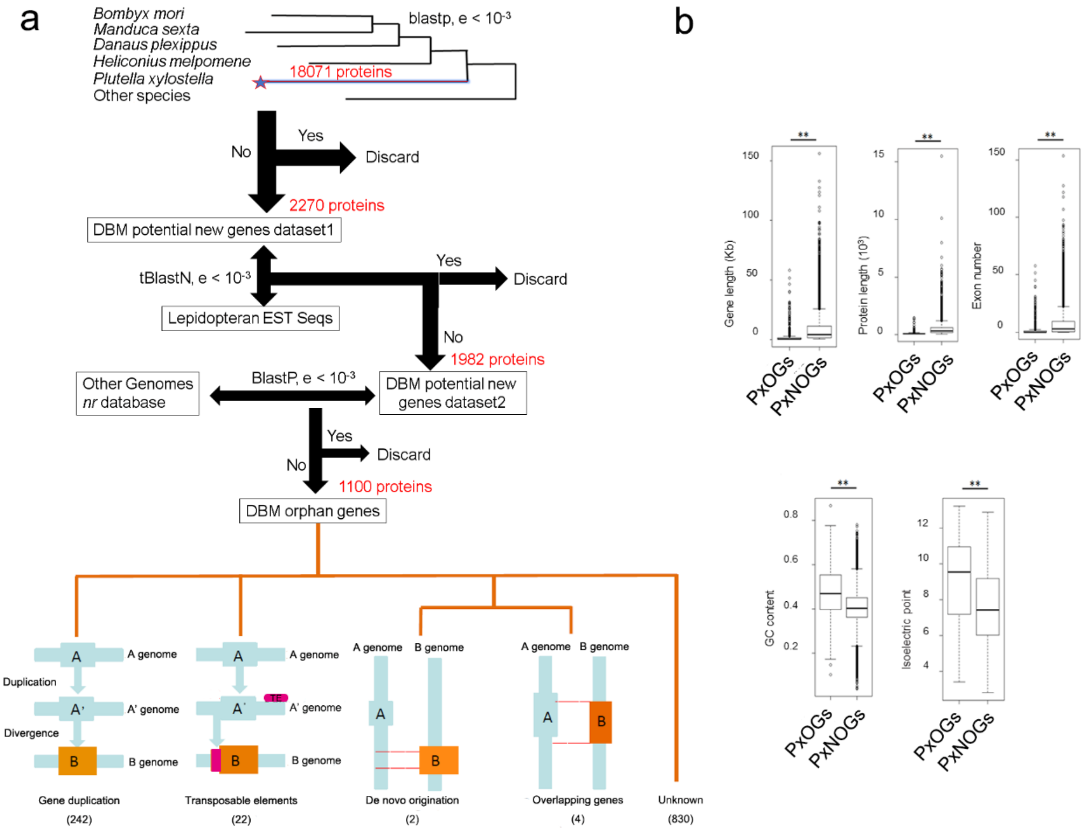

**Supplementary figure 2.** (a) Expression patterns of orphan genes based on RNA-Seq data. (b) Expression profiles of other six orphan genes validated by qRT-PCR. Expression level represents the relative expression level using *RIBP* as the control gene for qRT-PCR. The expression level is represented as the mean±SD (*n*=4) for qPCR analysis.

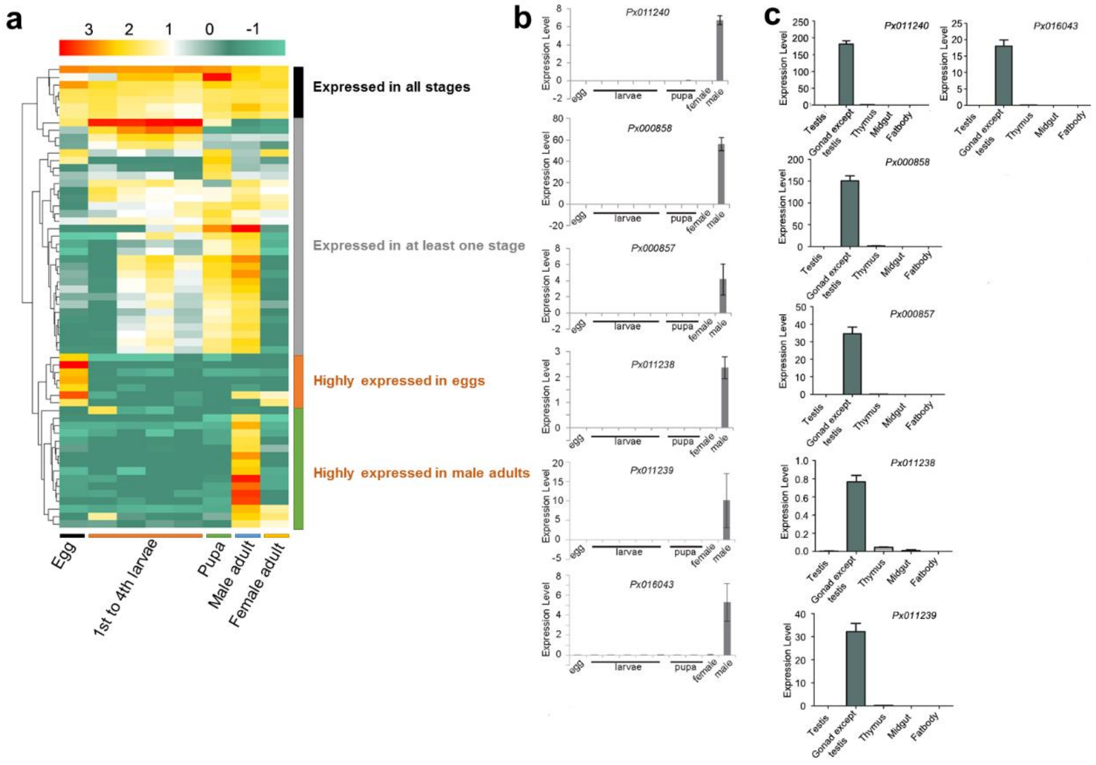

**Supplementary figure 3.** (a) The tissue expression profile of *lushu*. (b) Clone of *lushu* gene. (c) Gene structure of *lushu.* (d) Western blot analysis of Lushu in various tissues. SV denotes seminal vesicles, and MAG denotes male accessory glands. (e)Tissues of reproduction, silkgland and midgut from male G88 and lushu-null DBM strains were incubated with Lushu polyclonal antibody and HRP-Goat Anti-Rabbit IgG. We can only detect the Lushu (red) in reproduction tissues. (f) We can detect the Lushu on sperm bundles in vas deferens through immunofluorescence assays.

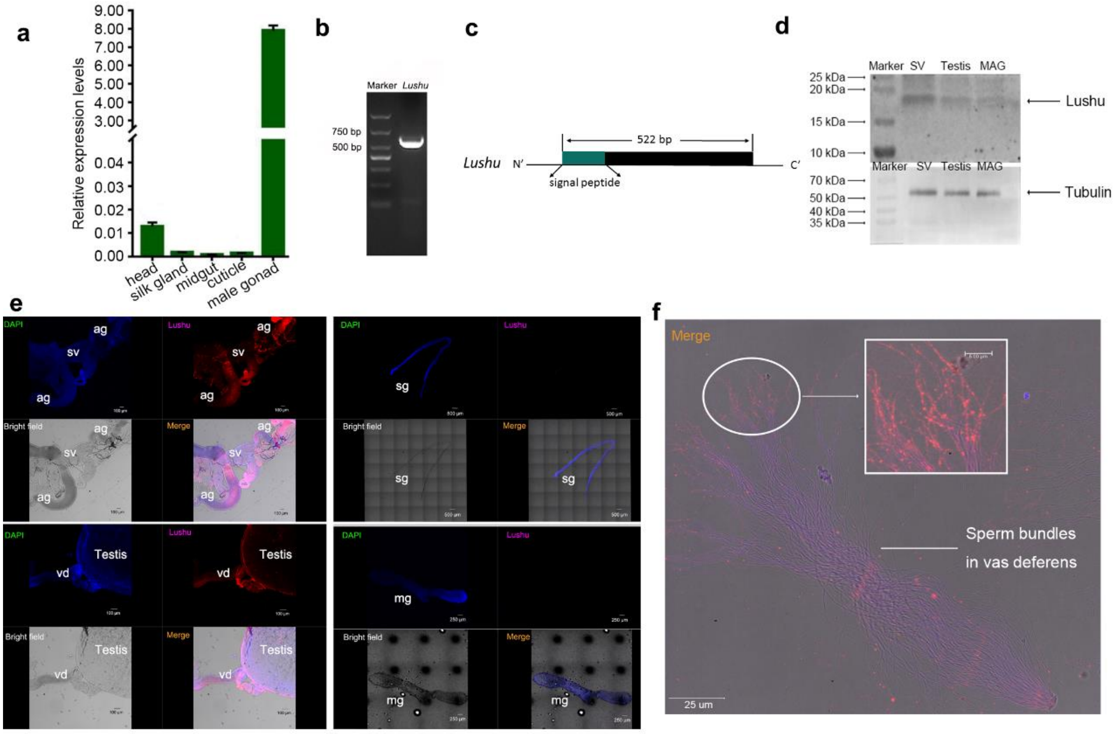

**Supplementary figure 4.** (a) Seminal vesicles from male abdomens. (b) Protein samples collected from 1d-old males were prepared and separated by 10% SDS-PAGE (lane 3). Then samples of total proteins were sent for LC-MS/MS analysis. (c) Pepetides specific to Lushu identified from sperm proteins collected from seminal vesicle and bursa of *G88* by LC-MS/MS. (d) Lushu protein sequences and the pepetides specific to Lushu. (e) The bursa were collected from *G88* females 30min after mating with *G88* males. (f) The seminal fluid proteins were collected from bursa and separated by 10% SDS-PAGE.

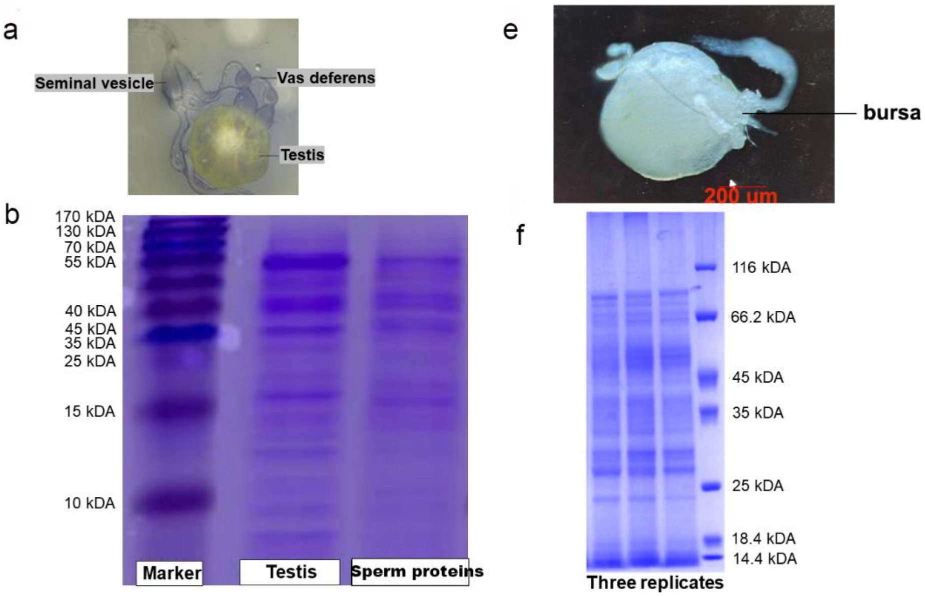


c Peptides specific to Lushu identified from proteome collected from seminal vesicles and bursa of G88 by LC-MS/MS

| G88 | Peptides | Total spectra | Total peptides |
|---|---|---|---|
| seminal vesicle | SGLGGVFR<br>LYAMSNSLFR<br>DIPAYGQQMPAR | 9 | 3 |
| bursa | LLKDNSGLDQHVTR<br>SGLGGVFR<br>YVVGKHDHPDSATNV<br>DIPAYGQQMPAR<br>DNSGLDQHVTR<br>HDHPDSATNV<br>DIPAYGQQMPARDNPAR | 7 | 7 |

d

>Lushu
MALFLLLSCVGLAAAATAAAAPPGPVQRPVEAGGGEDLAYRSGLGGVFRG
APGQWKQMLCSSNAGYDYAPPPMPYITSTYPQLPKNLVFTGPAGPMYTG
PQAFDPRLYAMSNSLFRGSQAFDRRLLKDNSGLDQHVTRDIPAYGQQMP
ARDNPARLMKYVVGKHDHPDSATNV

**Supplementary figure 5.** (a) Sperm protein samples from *G88* males and *lushu-null* males were prepared and separated by 10% SDS-PAGE. In order to identify the peptides with low abundance, gel sections containing proteins with molecular weights ranging from ~15 to 35 kDa were excised. (b) Peptides specific to Lushu identified from *G88* and *Lushu-null* by LC-MS/MS. (c) Tissues of reproduction from male *G88* and *lushu-null* strains were incubated with Lushu polyclonal antibody and HRP-Goat Anti-Rabbit IgG. We cannot detect the Lushu (red) in *lushu-null* males. (d) CRISPR-edited sites identified by whole genome re-sequencing.

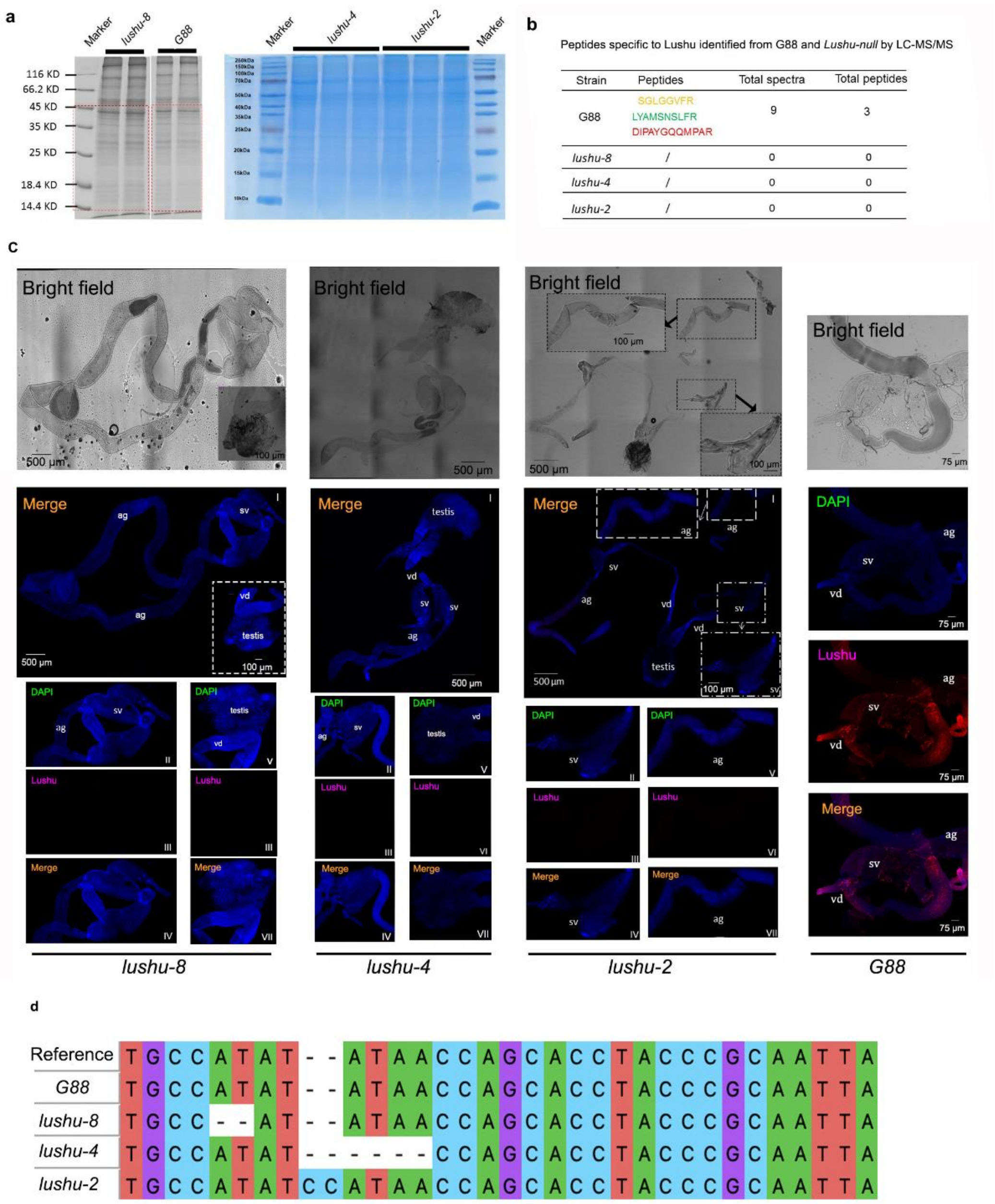

**Supplementary figure 6.** (a) Egg size comparison between *G88* males and *lushu-null* males. (b) Hatching rate comparison between *G88* males and *lushu-null* males under one-to-one mating. (c) (I-V) Fluorescence microscopic images of eupyrene sperm bundles in testIs of wildtype (*G88*) males and mutant males at 1d-old. Images represent the transformation from round spermatid cells through the elongation process to generate bundles of nearly mature spermatozoa.

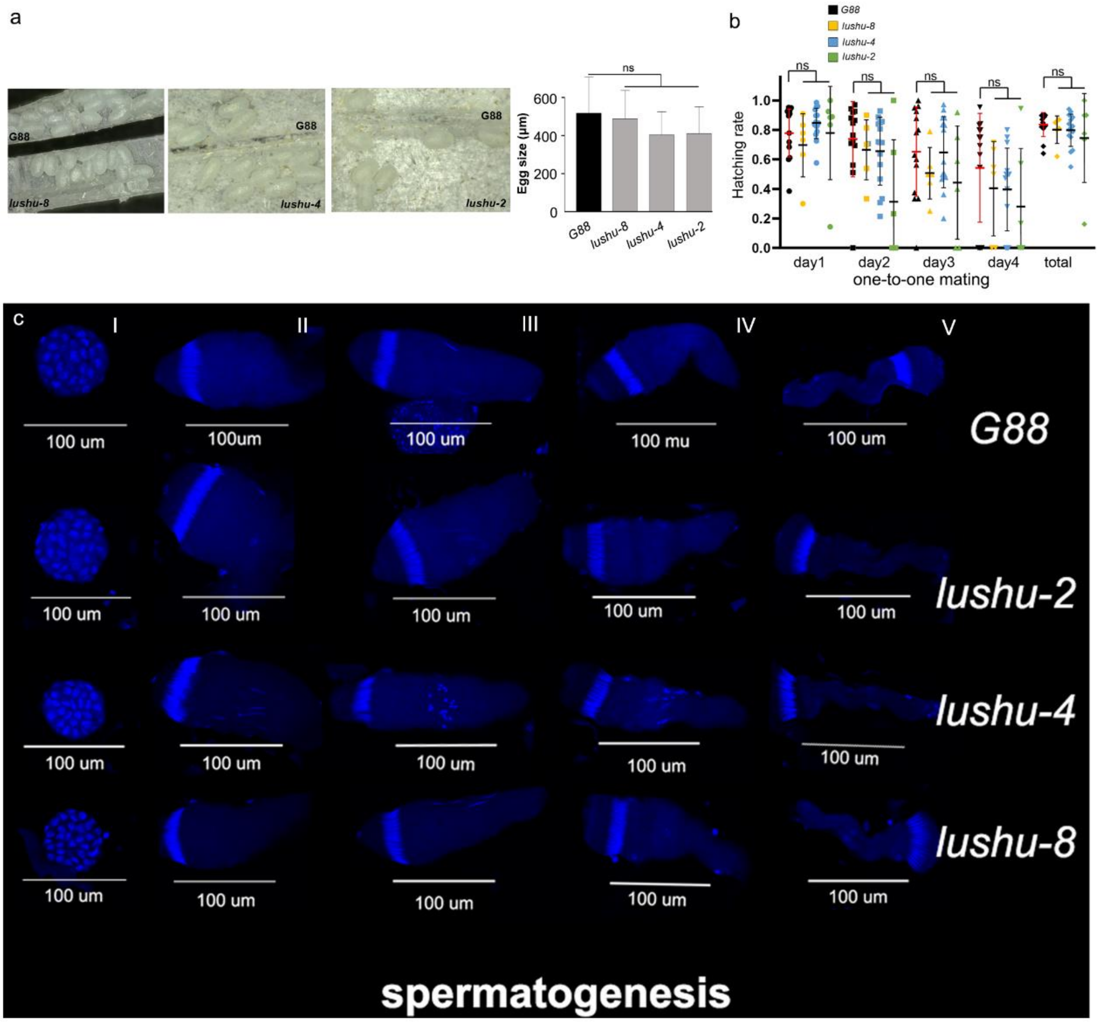

**Supplementary figure 7.** Life table of *G88* and *lushu-null* males*.* The term 'hours' refers to the developmental stages in hours.

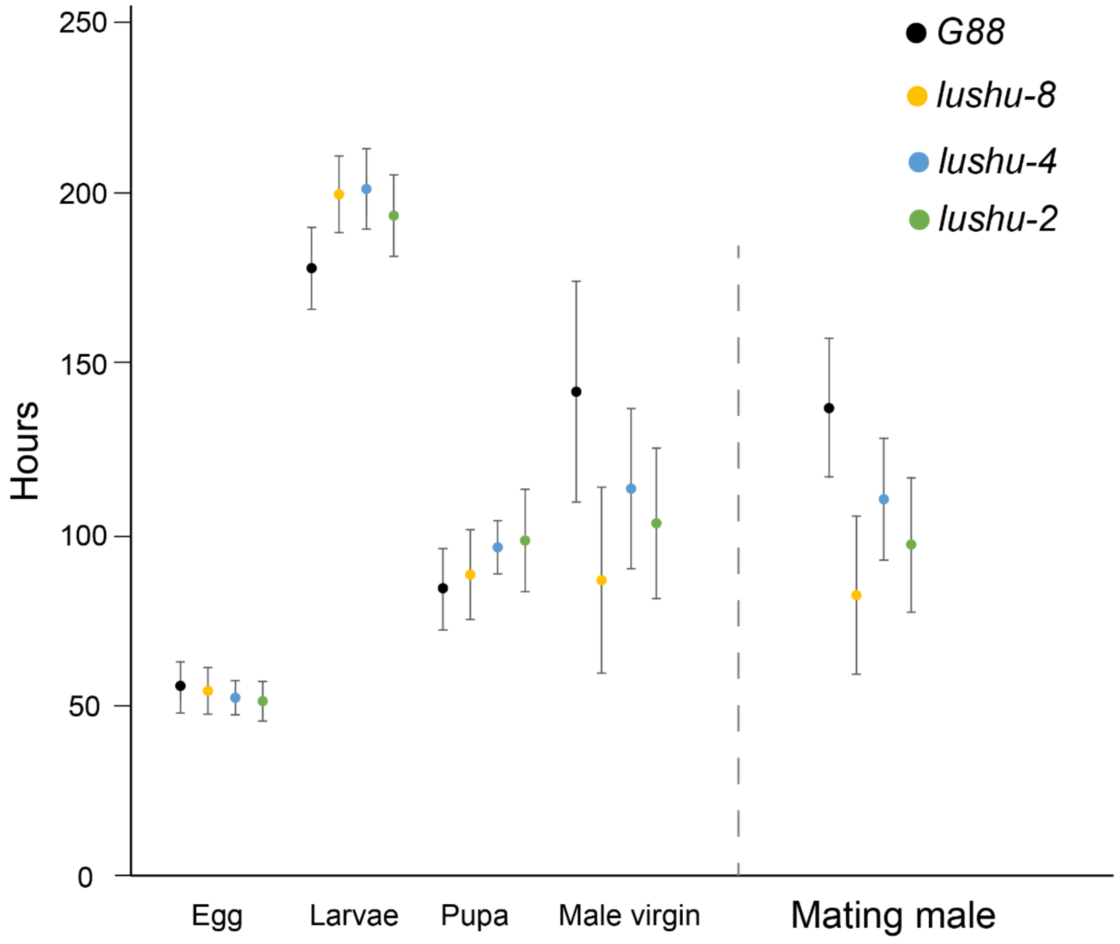

**Supplementary figure 8.** (a) Comparison of egg numbers fathered by *G88* and *lushu-null* males after 68 hours in the sperm competition assay. The egg number is represented as the mean ± SD (*n* = 4). Statistics analysis is performed using Student's t-test. (b) After mating once (30 minutes), we separate the males and females and record the total egg-laying capacity of the females. Statistics analysis is conducted using Student's t-test, with the egg number represented as the mean ± SD (*n* = 20). (c) After mating once (30 minutes), we separate the males and females and record the daily egg-laying capacity of the females. (d) Reproduction tissues comparison between *G88* and mutant males. (e) Comparison of sperm bundle retention ratio between wild-type and mutant males. The sperm bundle retention ratio was expressed as the mean±SD (*n* = 25). Statistical analysis was performing using Student's t-test.

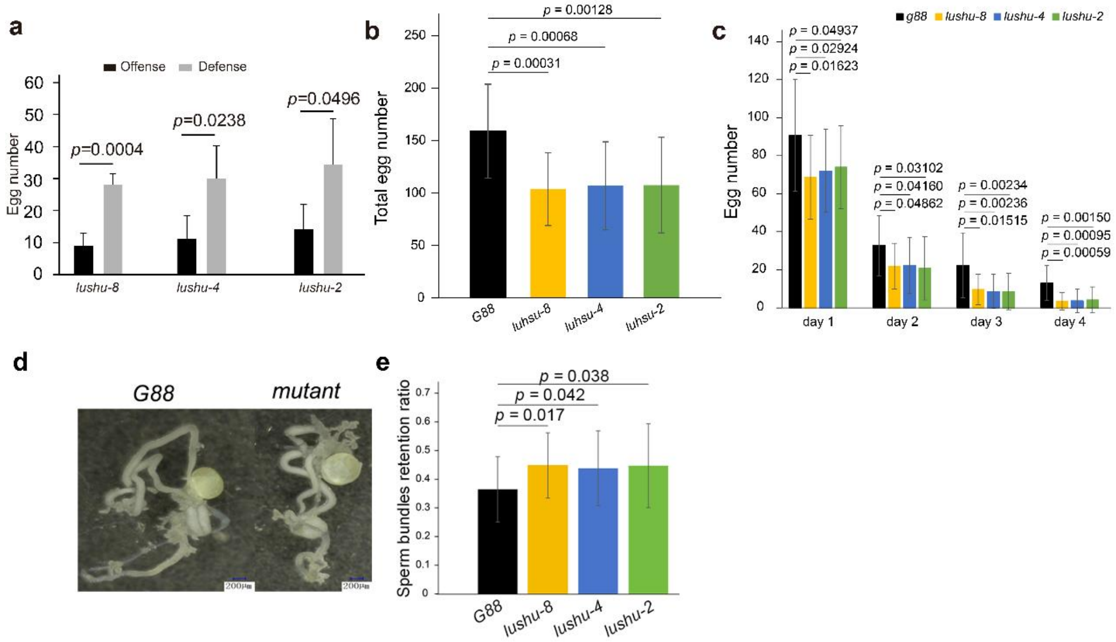

**Supplementary figure 9.** GO enrichment analysis of these DEGs between *G88* males and *lushu-8* males.

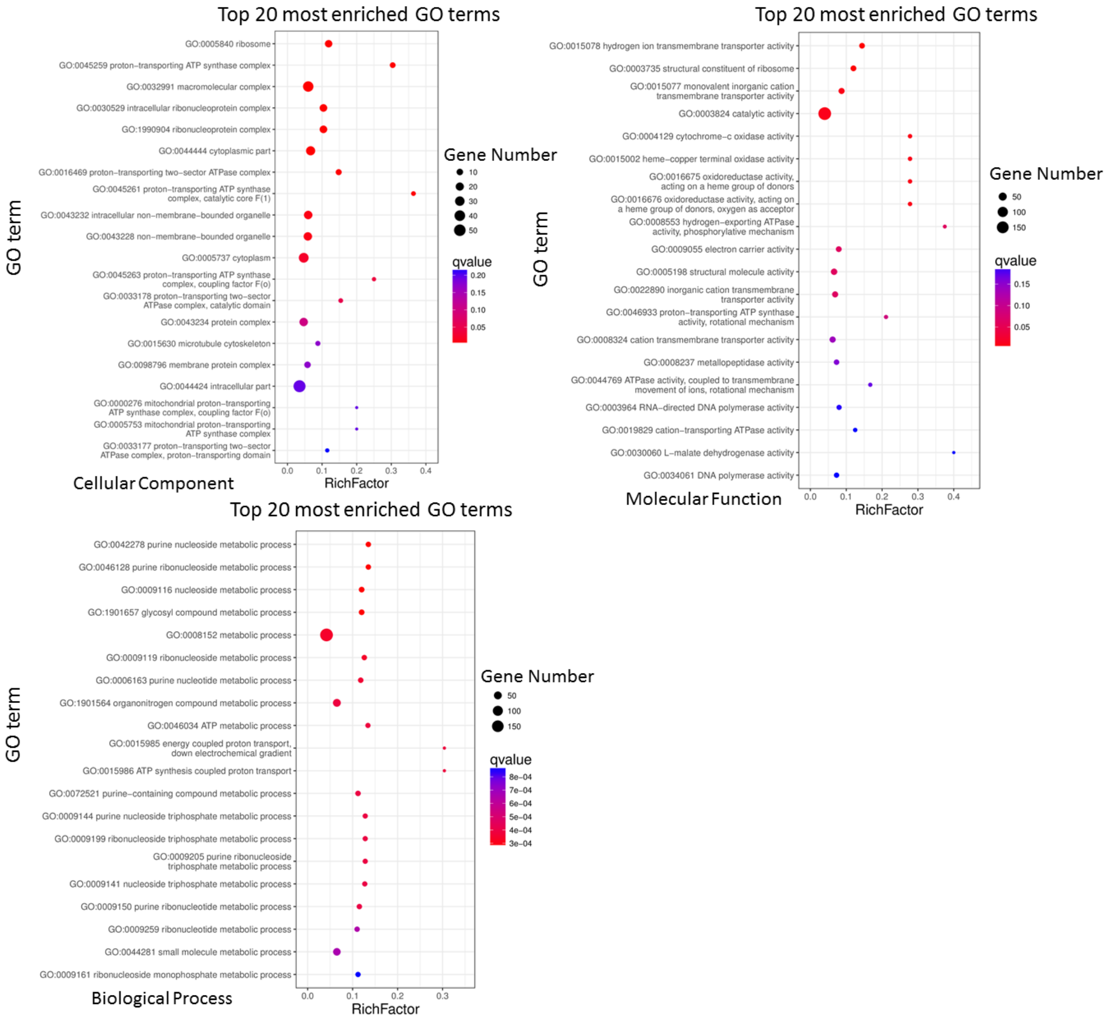

**Supplementary figure 10.** Top 20 most enriched KEGG pathways between *P. xylostella G88* males and *lushu-8* males.

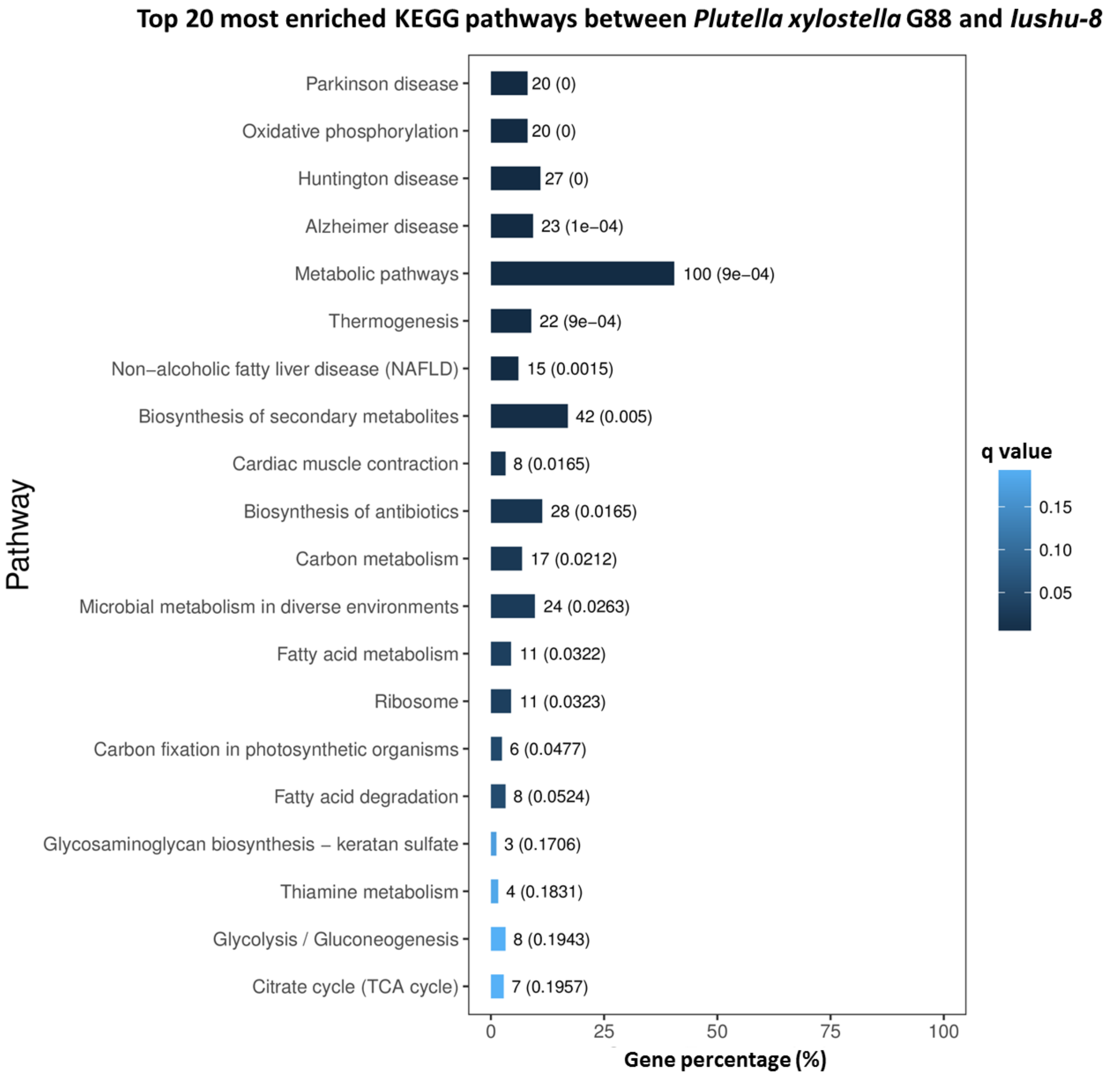

**Supplementary figure 11.** Insulin related pathways including glucose metabolism, TCA cycle and lipometabolism

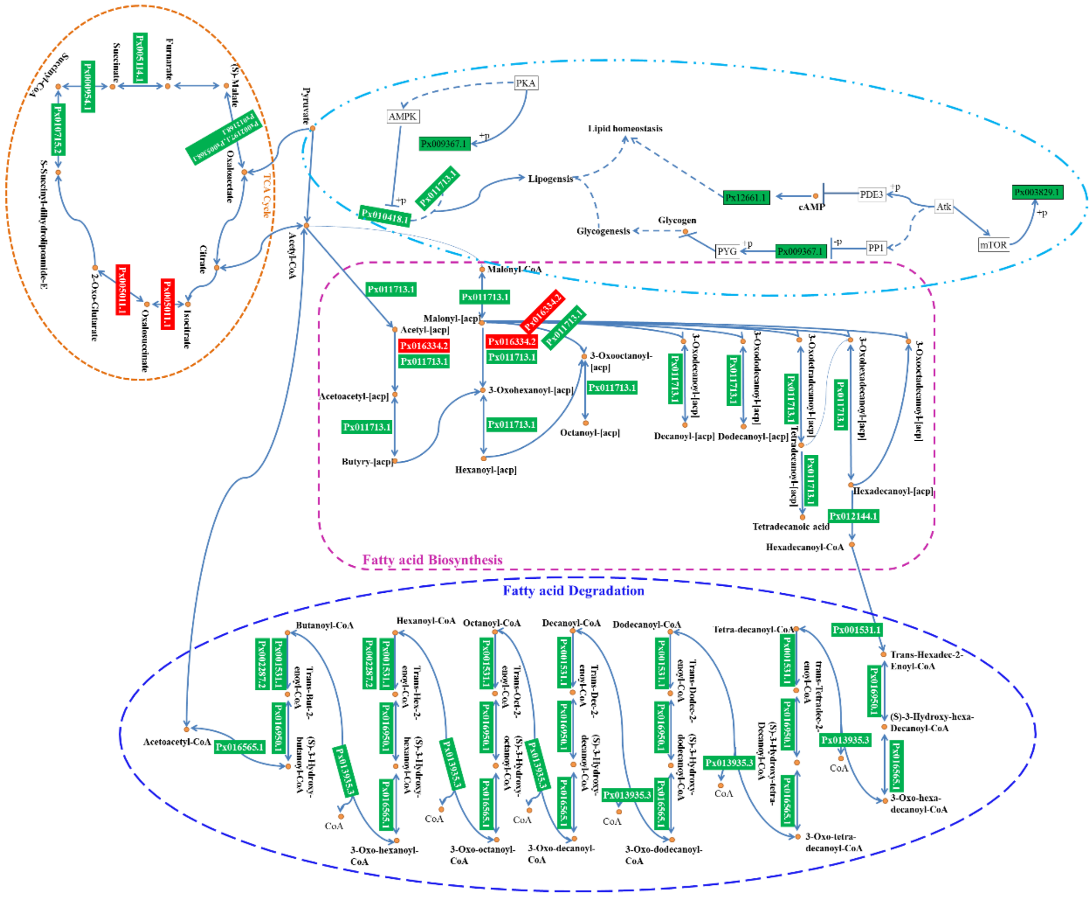

**Supplementary figure 12.** (a) Identification of *Lushu* sequences in different *P. xylostella* populations around world. (b) Synteny analysis of the scaffold containing *lushu* in *P. xylostella* was conducted in comparison with other closely related *Lepidoptera* organisms.

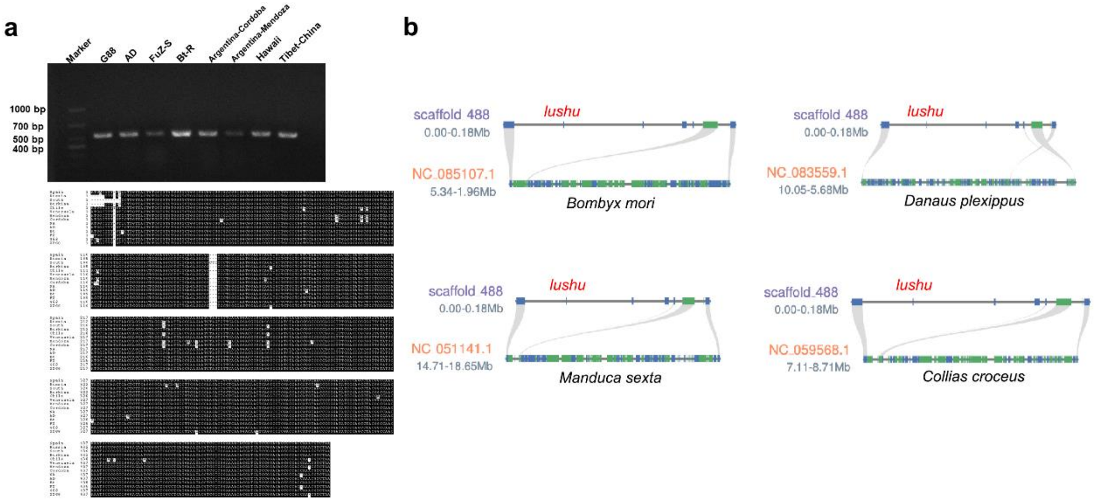

**Supplementary figure 13.** Homozygous mutant strain screening and establishment.

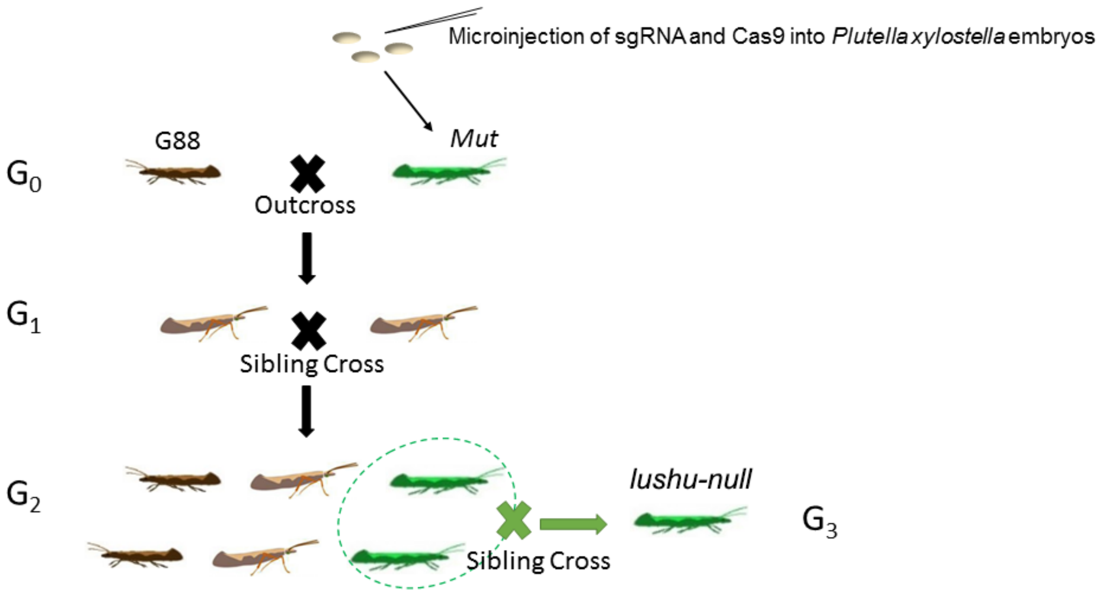


**Supplementary figure 14.** The off-target prediction pipeline.

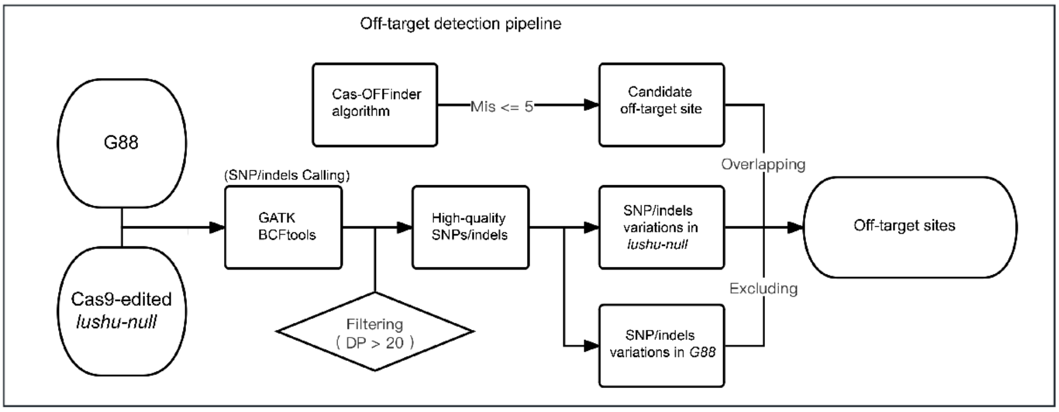

**Supplementary figure 15.** Standard curve of the ELISA assay to evaluate the ILP content.

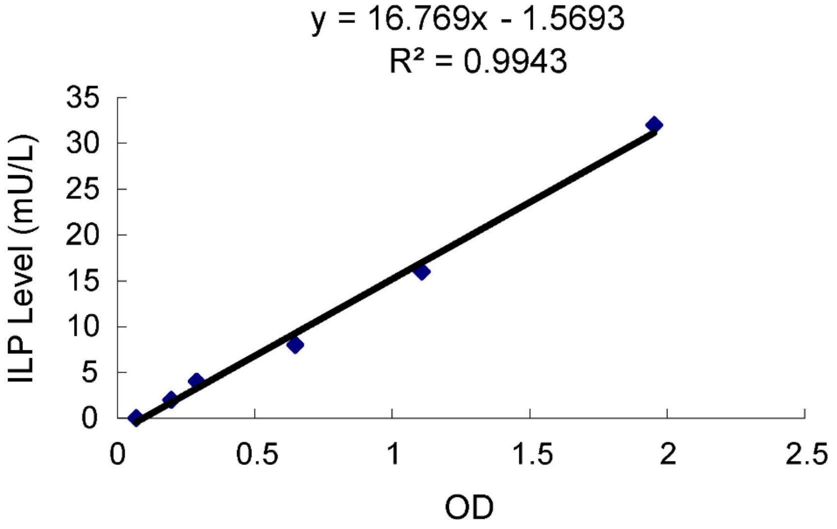

**Supplementary Table 1.** Orphan genes detected in the *P. xylostella* genome.

Listed in a separate Excel file.

**Supplementary Table 2.** Sperm proteins present in males collected from *P. xylostella.*

Listed in a separate Excel file.

**Supplementary Table 3.** Proteins present in three replicates of samples collected from spermatheca.

Listed in a separate Excel file.

**Supplementary Table 5.** Cas9-edited sites identification.

| | *lushu-8* | *lushu-4* | *lushu-2* |
|---|---|---|---|
| Overlap with predicted sites identified by Cas-OFFinder | 5 | 6 | 6 |
| Overlap with indels identified in G88 | 4 | 5 | 5 |
| Editing site | 1 | 1 | 1 |
| Off-target site | 0 | 0 | 0 |

**Supplementary Table 6.** Account for dead/living sperm cell in G88 and *lushu-null*.

| Insects | Splice | Total sperms | watchpoint | Sperms | Dead sperms |
|---|---|---|---|---|---|
| **G88** | 1 | 341 | 1 | 69 | 0 |
| | | | 2 | 63 | 0 |
| | | | 3 | 93 | 0 |
| | | | 4 | 54 | 0 |
| | | | 5 | 62 | 0 |
| | 2 | 172 | 1 | 20 | 0 |
| | | | 2 | 30 | 0 |
| | | | 3 | 39 | 0 |
| | | | 4 | 49 | 0 |
| | | | 5 | 34 | 0 |
| | 3 | 118 | 1 | 14 | 0 |
| | | | 2 | 24 | 0 |
| | | | 3 | 34 | 0 |
| | | | 4 | 27 | 0 |
| | | | 5 | 19 | 0 |
| | 4 | 143 | 1 | 26 | 0 |
| | | | 2 | 27 | 0 |
| | | | 3 | 32 | 0 |
| | | | 4 | 27 | 0 |
| | | | 5 | 31 | 0 |
| | 5 | 226 | 1 | 24 | 0 |
| | | | 2 | 44 | 0 |
| | | | 3 | 65 | 0 |
| | | | 4 | 60 | 0 |
| | | | 5 | 33 | 0 |
| | 6 | 250 | 1 | 60 | 0 |
| | | | 2 | 58 | 0 |
| | | | 3 | 48 | 0 |
| | | | 4 | 35 | 0 |

| | | | | | |
|---|---|---|---|---|---|
| | | | 5 | 49 | 0 |
| | 7 | 396 | 1 | 97 | 0 |
| | | | 2 | 61 | 0 |
| | | | 3 | 93 | 0 |
| | | | 4 | 65 | 0 |
| | | | 5 | 80 | 0 |
| | 8 | 263 | 1 | 60 | 0 |
| | | | 2 | 57 | 0 |
| | | | 3 | 51 | 0 |
| | | | 4 | 54 | 0 |
| | | | 5 | 41 | 0 |
| | 9 | 52 | 1 | 15 | 0 |
| | | | 2 | 6 | 0 |
| | | | 3 | 16 | 0 |
| | | | 4 | 13 | 0 |
| | | | 5 | 2 | 0 |
| ***lushu-8*** | 1 | 157 | 1 | 34 | 0 |
| | | | 2 | 35 | 0 |
| | | | 3 | 25 | 0 |
| | | | 4 | 31 | 0 |
| | | | 5 | 32 | 0 |
| | 2 | 201 | 1 | 42 | 0 |
| | | | 2 | 36 | 0 |
| | | | 3 | 39 | 0 |
| | | | 4 | 43 | 0 |
| | | | 5 | 41 | 0 |
| | 3 | 108 | 1 | 2 | 0 |
| | | | 2 | 27 | 0 |
| | | | 3 | 34 | 0 |
| | | | 4 | 20 | 0 |
| | | | 5 | 25 | 0 |
| | 4 | 163 | 1 | 53 | 0 |

| | | | | | |
|---|---|---|---|---|---|
| | | | 2 | 8 | 0 |
| | | | 3 | 27 | 0 |
| | | | 4 | 48 | 0 |
| | | | 5 | 27 | 0 |
| | 5 | 175 | 1 | 32 | 0 |
| | | | 2 | 38 | 0 |
| | | | 3 | 34 | 0 |
| | | | 4 | 39 | 0 |
| | | | 5 | 32 | 0 |
| | 6 | 191 | 1 | 36 | 0 |
| | | | 2 | 29 | 0 |
| | | | 3 | 41 | 0 |
| | | | 4 | 50 | 0 |
| | | | 5 | 35 | 0 |
| | 7 | 47 | 1 | 10 | 0 |
| | | | 2 | 9 | 0 |
| | | | 3 | 6 | 0 |
| | | | 4 | 14 | 0 |
| | | | 5 | 8 | 0 |
| ***lushu-4*** | 1 | 203 | 1 | 45 | 0 |
| | | | 2 | 60 | 0 |
| | | | 3 | 22 | 0 |
| | | | 4 | 35 | 0 |
| | | | 5 | 41 | 0 |
| | 2 | 170 | 1 | 35 | 0 |
| | | | 2 | 28 | 0 |
| | | | 3 | 45 | 0 |
| | | | 4 | 24 | 0 |
| | | | 5 | 38 | 0 |
| | 3 | 122 | 1 | 15 | 0 |
| | | | 2 | 18 | 0 |
| | | | 3 | 35 | 0 |

| | | | | | |
|---|---|---|---|---|---|
| | | | 4 | 24 | 0 |
| | | | 5 | 30 | 0 |
| | 4 | 146 | 1 | 48 | 0 |
| | | | 2 | 5 | 0 |
| | | | 3 | 25 | 0 |
| | | | 4 | 42 | 0 |
| | | | 5 | 26 | 0 |
| | 5 | 168 | 1 | 31 | 0 |
| | | | 2 | 28 | 0 |
| | | | 3 | 35 | 0 |
| | | | 4 | 41 | 0 |
| | | | 5 | 33 | 0 |
| ***lushu-2*** | 1 | 192 | 1 | 31 | 0 |
| | | | 2 | 55 | 0 |
| | | | 3 | 23 | 0 |
| | | | 4 | 37 | 0 |
| | | | 5 | 46 | 0 |
| | 2 | 164 | 1 | 35 | 0 |
| | | | 2 | 22 | 0 |
| | | | 3 | 44 | 0 |
| | | | 4 | 20 | 0 |
| | | | 5 | 43 | 0 |
| | 3 | 116 | 1 | 11 | 0 |
| | | | 2 | 24 | 0 |
| | | | 3 | 14 | 0 |
| | | | 4 | 31 | 0 |
| | | | 5 | 36 | 0 |
| | 4 | 120 | 1 | 42 | 0 |
| | | | 2 | 41 | 0 |
| | | | 3 | 11 | 0 |
| | | | 4 | 14 | 0 |
| | | | 5 | 12 | 0 |

| | | | | |
|---|---|---|---|---|
| 5 | 162 | 1 | 29 | 0 |
| | | 2 | 28 | 0 |
| | | 3 | 33 | 0 |
| | | 4 | 43 | 0 |
| | | 5 | 29 | 0 |

**Supplementary Table 7.** Genes exhibiting significantly different transcript abundances between males of *G88* and *lushu-8*.

Listed in a separate Excel file.

**Supplementary Table 8.** Significant GO terms for DEGs between *G88* and *lushu-8* of *P. xylostella.*

Listed in a separate Excel file.

**Supplementary Table 9.** Sequences of primers used to amplify the *Lushu* gene from *P. xylostella* strains.

| Gene | Primer Sequences (5’ – 3’) | Direction | Application |
|---|---|---|---|
| *Lushu* | ATGGCTTTGTTCTTGTTACTG | + | DNA PCR amplification of full-length *Lushu* CSD |
| | TTAGACGTTGGTGGCGCTGTC | - | |
| | GTTACTGTCGTGTGTGGG | + | qRT-PCR for expression patterns analysis |
| | GGATGATCGTGTTTTCCG | - | |
| | GATGCTCTGCTCGTCTAATGCGG | + | Genomic copy number evaluation |
| | GCCCTGCGGGTCCTGTGAACACT | - | |
| *PxyMasc* | GGTACCACCTCAGTGCCTCATC | + | Z-linked genes in *P. xylostella* |
| | AACTATGTGACTTACTGGGCCGA | - | |
| *kettin* | TACAGCCAGCTCGCGAATC | + | Z-linked genes in *P. xylostella* |
| | GCCCGTAGGTGCATGATGTT | - | |

**Supplementary Table 10.** Sequence of sgRNA targeting *Lushu* in *P.xylostella*.

| sgRNA | Target position started | Sequence (5’ – 3’) | Application |
|---|---|---|---|
| *Lushu*-sg2 | 138 bp | CGTGTTCAGAGGCGCTCC | sgRNA target sequence for *Lushu* |

**Supplementary Table 11.** Targeted mutagenesis of *Lushu* in *P. xylostella* using CRISPR/Cas9.

| Times | Strain | Target gene | Cas9 source | sgRNA | Hatchability | Survival rate | Mosaic $G_0$ |
|---|---|---|---|---|---|---|---|
| 1 | G88 | *Lushu* | mRNA | 5'-GGGTAGGTGCTGGTTATATATGG-3' | 32.9% (51/155) | 60% (31/51) | 62.5% (5/8) |
| 2 | G88 | *Lushu* | mRNA | 5'-GGGTAGGTGCTGGTTATATATGG-3' | 44.2% (42/95) | 69% (29/42) | 54.5% (6/11) |
| 3 | G88 | *Lushu* | mRNA | 5'-GGGTAGGTGCTGGTTATATATGG-3' | 46.7% (56/120) | 45% (25/56) | 64.3% (9/14) |

Hatchability = number of hatched neonates/number of embryos injected.

Survival rate = number of adults/number of hatched neonates.

Mosaic $G_0$ = number of mutated $G_0$ moths detected/number of $G_0$ moths randomly detected.

**Supplementary Table 12.** Sequences of primers used to detect the mutation of *Lushu*.

| Region | Primer Sequences (5' – 3') | Direction | Application |
|---|---|---|---|
| *Lushu* | ACTGTCGTGTGTGGGTCTGGC | + | Allele specific PCR detection for mutation |
| | TGTCCGGATGATCGTGTTTTC | - | |

**Supplementary Table 13. Statistics of genomic re-sequencing data.**

| | Total reads | Mapping rate | Depth | SNPs | Indels |
|---|---|---|---|---|---|
| *lushu-8* | 18,242,004 | 0.92 | 6.86 | 68,576 | 5,461 |
| *lushu-4* | 20,130,782 | 0.93 | 7.53 | 47,898 | 5,422 |
| *lushu-2* | 16,979,186 | 0.92 | 6.41 | 51,311 | 4,346 |
| *G88* | 18,445,184 | 0.92 | 6.74 | 73,308 | 6,332 |

**Supplementary Table 14.** RNA-Seq data from *G88* and *lushu-8* of *P. xylostella.*

| Sample | *G88-1* | *G88-2* | *G88-3* | *Lushu-8-1* | *Lushu-8-2* | *Lushu-8-3* |
|---|---|---|---|---|---|---|
| Raw reads (number) | 31,459,110 | 34,335,756 | 29,964,700 | 32,073,392 | 34,551,786 | 35,318,360 |
| Raw Bases (number) | 4,718,866,500 | 5,150,363,400 | 4,494,705,000 | 4,811,008,800 | 5,182,767,900 | 5,297,754,000 |
| Clean Reads (number) | 30,868,428 | 33,263,724 | 29,150,476 | 31,525,130 | 33,999,512 | 34,685,260 |
| Clean Reads (%) | 98.12 | 96.88 | 97.28 | 98.29 | 98.4 | 98.21 |
| Clean Bases (number) | 4,630,264,200 | 4,989,558,600 | 4,372,571,400 | 4,728,769,500 | 5,099,926,800 | 5,202,789,000 |
| Low-quality Reads (number) | 206,866 | 360,580 | 189,074 | 198,582 | 176,866 | 222,708 |
| Low-quality Reads Rate (%) | 0.66 | 1.05 | 0.63 | 0.62 | 0.51 | 0.63 |
| Adapter-contaminated Reads | 348,834 | 659,664 | 593,714 | 316,238 | 345,096 | 371,320 |
| Adapter-contaminated Reads Rate (%) | 1.11 | 1.92 | 1.98 | 0.99 | 1 | 1.05 |
| Raw Q30 Bases (%) | 91.6 | 91.47 | 91.08 | 92.41 | 92.3 | 91.65 |

**Supplementary Table 15.** Primers used to verify the expression of candidate genes in various metabolic pathways in which *lushu* might be related with.

| Gene | Primer sequences (5' – 3') | Direction | Pathways |
|---|---|---|---|
| Px009367 | GAGAGAGAAGCCGAAGAAGTTT | F | Insulin signaling pathway |
| | ATGGCTCCACGGATGTTTAG | R | |
| Px012661 | CTAGTGCTAGACGCGATGTTC | F | |
| | TCCGTTCTCGATGACGTAGA | R | |
| Px010418 | AGCTCCTCGTCTCACTCATTA | F | |
| | TTGCACCCAGTCCACTTTAC | R | |
| Px011713 | GGAGGTCAAGCCTGTGTTTAT | F | |
| | GCATTGCAGCCCGTAGA | R | |
| Px003829 | GAGGTTAAACAGTCGGAACTACA | F | |
| | CTAGCCTCACGAAGGTCTTTAC | R | |
| Px012832 | GTCAACACTCCTGTGTCAGATAG | F | Galactose metabolism |
| | AGGAAATGGCCTGGTGTTAG | R | |
| Px001761 | GTGCTGTGGTAGATGGTGATAAA | F | |
| | GGCGAGGTACTGACTCTGATTA | R | |
| Px008360 | TGTTGTCATTCGTCGGGATAAG | F | |
| | CACTTGTAGTGGCGAGAATAGG | R | |
| Px001933 | CTGTGCGACCACTACATACTT | F | |
| | CAGAAAGCGTCCAGGGTTAT | R | |
| Px005011 | CCGCTGTATTTATCCACCAAGA | F | TCA cycle |
| | CTGCTTCTTGTAGTCCTTCTGG | R | |
| Px005114 | GTCAACAAGCCCTCCAAGAT | F | |
| | GGCTCAATGGACTGGTACTG | R | |
| Px002197 | AGAATATACCGCCGGGTTTG | F | |
| | TTAGCTGCCCACACTTGTATAG | R | |
| Px010715 | CGAGTTTAAGAGCACACGGTAT | F | |
| | CGGGTCACCTTCCTTCTTTATT | R | |
| Px001531 | AATTTCCTTGACGGAGGAGTG | F | Fatty acid degradation |
| | CTTCAGGGATGGAATCGAAGAG | R | |
| Px016565 | GCACCAGCTCACACTATCTT | F | |
| | GAGGCCGCCGAATTTATCT | R | |
| Px016950 | GACAAGGATGAGAAGGCTAAGG | F | |
| | AGCATCGAATAGGGCATTGTAG | R | |
| Px002287 | TGGATGTCGCTGTGGAATAC | F | |
| | TCTGACAGCTTGCTCTGAATAG | R | |
| Px011713 | GGAGGTCAAGCCTGTGTTTAT | F | Fatty acid biosynthesis |
| | GCATTGCAGCCCGTAGA | R | |
| Px016334 | CCTGCCACAGGATTAGCATTAG | F | |
| | GTGGCTTCTCTGTCTTCATCAG | R | |
